# Gene–Diet Interactions in Type 2 Diabetes: A Systematic Review of Observational Studies and Clinical Trials

**DOI:** 10.3390/nu18152439

**Published:** 2026-07-26

**Authors:** Angeliki Kapellou, Sevastiani Papailia, Thanasis Fotis, Dimitrios Miltiadis Vrachnos, Dimitrios Papageorgiou, Effie Salata, Eleni Ntoumou, Spiros Vittas

**Affiliations:** 1 iDNA Laboratories, 7 Kavalieratou Taki, 14564 Kifissia, Greece; angie.kapellou@idna.gr (A.K.); sevastiani.papailia@idna.gr (S.P.); athanasios.fotis@idna.gr (T.F.); dimitris.vrachnos@idna.gr (D.M.V.); dpapageorgiou@mit.gov.tr (D.P.); effie.salata@idna.gr (E.S.); eleni.ntoumou@idna.gr (E.N.); 2 Department of Materials Science and Engineering, Massachusetts Institute of Technology, Cambridge, MA 02139, USA

**Keywords:** nutrigenetics, precision nutrition, polymorphisms, dietary patterns, glycemic control, personalized nutrition

## Abstract

Background/Objectives: Type 2 diabetes (T2D) is a multifactorial disorder arising from complex interactions between genetic susceptibility and environmental exposures, including diet. This systematic review aimed to synthesize and critically evaluate evidence on gene–diet interactions in T2D-related phenotypes among adults without diagnosed T2D at baseline. Methods: The review was conducted according to PRISMA guidelines and registered in PROSPERO (CRD420251175793). PubMed and Embase were systematically searched for eligible studies published up to November 2025. Observational studies and intervention trials examining interactions between dietary exposures and genetic variants, haplotypes, or genetic risk scores (GRSs) in relation to incident T2D, insulin resistance, glycemic traits, or β-cell function were included. Risk of bias was assessed using study design-specific tools. Results: A total of 61 reports representing 27 unique studies met the eligibility criteria, comprising 30 reports of randomized trials and 31 reports of non-randomized studies. Across studies, 55 Single Nucleotide Polymorphisms (SNPs) in 40 genes were examined, while 11 studies evaluated GRSs. The most frequently studied loci included *TCF7L2*, *ADIPOQ*, *FTO*, *IRS1*, *GIPR* and *PPM1K*. Reported interactions involved dietary patterns, macronutrient composition, dietary fat quality, fiber/whole-grain intake and specific foods, influencing incident T2D, insulin-related traits and glycemic outcomes. However, findings were heterogeneous, frequently inconsistent, and often lacked independent replication. Most randomized and non-randomized studies were judged at high or serious risk of bias. Conclusions: Genetic variation may contribute to interindividual differences in metabolic responses to diet, although robust and reproducible gene–diet interactions remain limited. Larger, well-powered, standardized studies across diverse populations are needed to establish clinically meaningful applications of Precision Nutrition (PN) in T2D prevention.

## 1. Introduction

Type 2 diabetes (T2D) is a highly prevalent, multifactorial, and heritable metabolic disorder characterized by a complex and heterogeneous genetic architecture [1,2]. It represents a major and growing global public health challenge, with approximately one in nine adults—nearly 589 million people—currently living with diabetes, of which around 90% of cases are T2D [2,3]. By 2045, this number is projected to reach 700 million [4]. Although T2D predominantly affects adults over 50 years of age, its incidence is rising among children, adolescents, and young adults [5].

From a pathophysiological perspective, T2D is defined by impaired insulin secretion due to pancreatic β-cell dysfunction and reduced tissue responsiveness to insulin. In early disease stages, compensatory hyperinsulinemia partially preserves glucose homeostasis; however, progressive β-cell failure ultimately leads to overt T2D. Functionally, β-cell dysfunction is reflected in a blunted first-phase postprandial insulin response and prolonged postprandial hyperinsulinemia [6,7]. Impaired glucose tolerance (IGT), an intermediate state between normoglycemia and diabetes, is marked by elevated blood glucose concentrations and confers substantial risk for progression to overt T2D, with up to 70% of affected individuals developing diabetes and an annual conversion rate of 5–10% [8,9].

Beyond metabolic dysregulation, T2D is increasingly recognized as a chronic low-grade inflammatory condition. Inflammation contributes to insulin resistance and β-cell dysfunction, while hyperglycemia and broader metabolic disturbances further amplify inflammatory processes [10]. Consequently, T2D is associated with both microvascular and macrovascular complications, notably retinopathy, neuropathy, nephropathy, and atherosclerotic cardiovascular disease [2]. Individuals with T2D have a two- to three-fold higher risk of atherosclerotic cardiovascular disease [11] and life expectancy is reduced by seven years for men and approximately six years for women [12].

T2D arises from a complex interplay between genetic susceptibility and environmental exposures. Major contributing factors include obesity, physical inactivity, energy-dense and nutritionally poor dietary patterns, chronic stress, and family history [13,14]. Ethnicity also influences disease onset and progression, with White and Asian populations showing lower age-adjusted prevalence than Hispanic and Black populations [15]. At the genetic level, T2D is associated with variation in pathways regulating β-cell function and insulin secretion (e.g., *KCNJ11*), insulin signaling and action (e.g., *IRS1*, *PPARG*, and *FTO*), appetite and energy homeostasis (e.g., *MC4R*), and transcriptional control of glucose metabolism (e.g., *TCF7L2*) [6,13]. By 2022, multi-ancestry genome-wide association studies (GWAS) had identified more than 700 loci associated with T2D; however, these account for only around 20% of disease heritability, underscoring the problem of “missing heritability,” as individual common variants generally confer only modest effects on risk (approximately 10–30%) [1,16]. In contrast, family history remains a strong predictor of T2D, with heritability estimates ranging from 20% to 80% [17]. The risk of developing T2D is approximately two- to three-fold higher in individuals with an affected first-degree relative and five- to six-fold higher in those with both maternal and paternal family history [18].

Dietary and lifestyle modifications, particularly weight management, can prevent or delay T2D onset, with physical activity and healthy dietary interventions reducing risk by up to 47%. Protective patterns such as the Mediterranean diet and the Dietary Approaches to Stop Hypertension (DASH) are rich in whole grains, fiber, and low-fat dairy, whereas high glycemic load diets high in added sugars and red/processed meat are associated with increased risk [19,20]. Nevertheless, responses to dietary interventions vary substantially between individuals, and personalized approaches may yield differing degrees of glycemic and metabolic improvement compared with standard recommendations [21]. This heterogeneity underscores the importance of individual genetic architecture and gene–diet interactions in T2D. Genetic variants and dietary exposures may converge on shared molecular pathways involved in glucose metabolism, insulin sensitivity, and inflammation, generating effects that are not simply additive [3,22].

Understanding gene–diet interactions in T2D is, therefore, critical for advancing evidence-based, personalized dietary prevention strategies. Historically, progress in this field was limited by methodological constraints, including incomplete characterization of genetic risk and reliance on multiplicative interaction models, which may have obscured genotype-specific dietary responses. Advances in Nutrigenetics and Precision Nutrition (PN) over the past decade have addressed several of these limitations [23,24]. However, clinical implementation remains limited due to methodological heterogeneity, technical constraints, insufficient clinical trial data, limited population diversity, and practical barriers to translation [25,26,27].

Previous reviews have summarized the biological rationale and emerging evidence supporting PN approaches in diabetes prevention and management, including a recent narrative review of gene–diet interactions [28] and a systematic review of gene–lifestyle interactions in relation to T2D incidence [29]. However, previous syntheses have primarily focused on T2D risk, specific lifestyle factors, or broader diabetes populations. A systematic evaluation focusing on adults without diagnosed T2D at baseline and integrating evidence from both observational studies and intervention trials remains limited. Therefore, this systematic review aimed to synthesize and critically evaluate the existing evidence on gene–diet interactions in this population, with an emphasis on PN and metabolic outcomes.

## 2. Materials and Methods

### 2.1. Search Strategy

This systematic review was conducted in accordance with the Preferred Reporting Items for Systematic Review and Meta-Analyses (PRISMA) guidelines and was registered in PROSPERO (CRD420251175793). A comprehensive literature search was independently performed by two investigators in PubMed and Embase to identify relevant studies published up to 30 November 2025. The search strategy was developed by consensus and combined Medical Subject Headings (MeSH) and free-text keywords related to type 2 diabetes, gene–diet interactions, genetic variation, dietary exposures and relevant study designs. The complete database-specific search strategies, including search terms, Boolean operators, field tags and applied limits, are provided in Supplementary Table S1.

All retrieved records were imported into Rayyan (Rayyan Systems Inc., Doha, Qatar; web version, accessed 20 December 2025), a web-based platform designed for systematic review management [30]. Duplicate entries were removed, followed by title and abstract screening. In addition, the reference lists of all included studies and relevant review articles were manually screened, and forward citation tracking was performed to identify additional eligible studies.

### 2.2. Study Selection

Two investigators independently screened all records for inclusion in the systematic review using a two-step process: first by reviewing titles and abstracts, and then by evaluating full-text articles, according to predefined inclusion and exclusion criteria. Eligibility was assessed using the Population, Intervention, Comparator, Outcome, and Study design (PICOS) framework [31] (Table 1).

Primary outcomes of interest were the incidence or risk of T2D, measures of insulin resistance such as the Homeostatic Model Assessment for Insulin Resistance (HOMA-IR), and indicators of glucose homeostasis, including fasting glucose, fasting insulin, glycated hemoglobin (HbA1c), and results from oral glucose tolerance tests (OGTT). Secondary outcomes included metabolic biomarkers relevant to glycemic control. Although case–control, nested case–control, and case–cohort studies were eligible, only analyses relevant to adults without diagnosed T2D at baseline or those evaluating incident T2D risk among individuals free of diagnosed T2D at baseline, as well as intermediate glycaemic phenotypes prior to diagnosis, were considered for inclusion. Both observational and interventional human studies were eligible for inclusion, specifically cohort, case–control, and cross-sectional studies that reported interaction terms, as well as randomized and non-randomized clinical trials. Studies were excluded if they were conducted in non-human or in vitro models, if written in a language other than English, or were review articles, letters, case reports, or study protocols. Furthermore, studies were excluded if they lacked either formal interaction testing or stratified analyses that allowed for the evaluation of gene–nutrient interactions.

There were no restrictions placed on the publication date. Only full-text, peer-reviewed articles were included in the final analysis. Gray literature (including conference abstracts, dissertations, trial registries and preprints) was not searched or considered for inclusion. Investigators were blinded to each other’s decisions during the screening process, and any discrepancies were resolved by consensus following independent assessments.

### 2.3. Data Extraction

In this systematic review, outcome data were extracted exclusively from participants for whom both genetic information and data on nutrient intake or supplementation were available. Two investigators independently performed the data extraction process, and any discrepancies were resolved through consensus. For each included study, the following information was extracted: first author’s name, year of publication, study design, participant characteristics (including sample size, sex, age and intervention details), the genetic variant(s) investigated and the primary and secondary outcomes, along with corresponding statistical findings.

When studies reported multiple outcomes, dietary exposures, genetic variants, statistical models, or follow-up time points, data were extracted for all gene–diet interactions relevant to the predefined outcomes of interest and reported in the review. For studies with repeated measurements, results from the primary intervention time point or main analysis were extracted, while additional follow-up findings were included where they provided clinically relevant information. When multiple analyses were available within a study, data extraction followed a predefined hierarchy prioritizing the prespecified primary outcome, the primary intervention or follow-up time point, the most fully adjusted statistical model, and formal tests of gene–diet interaction. Where available, multiplicity-adjusted results were preferentially extracted. This approach was applied consistently across all included studies to minimize selective emphasis during data extraction.

All extracted data were systematically organized and grouped according to the reported study outcomes (incident T2D risk, insulin-related traits, and glycemic traits/β-cell function). This outcome-based approach was selected to facilitate comparison of gene–diet interactions across clinically related metabolic endpoints. Within each outcome category, evidence from observational and intervention studies was synthesized narratively, while study design, methodological characteristics, and risk-of-bias assessments were explicitly reported and considered during interpretation of the findings. This approach enabled the integration of evidence across clinically related outcomes while acknowledging the differing methodological strengths and limitations of the included study designs.

### 2.4. Risk of Bias Assessment

The risk of bias in the included studies was assessed independently by two investigators following the Cochrane Review guidelines [32] and conflicts were resolved by consensus. For randomized controlled trials, the Revised Cochrane risk-of-bias tool for randomized trials (RoB 2) was applied. The appropriate version—either the parallel-group or crossover RoB 2 tool—was selected based on study design. Each trial was rated at the domain level and overall, as having ‘low risk’, ‘some concerns’, or ‘high risk’ of bias [33].

For non-randomized studies, the ROBINS-I (Risk Of Bias In Non-randomized Studies of Interventions) tool was used. Although ROBINS-I was originally developed for non-randomized studies of interventions, its conceptual framework is based on the emulation of a hypothetical target trial and has been applied in systematic reviews, including heterogeneous non-randomized study designs [32]. In the context of nutritional epidemiology, habitual dietary exposures can be conceptualized as modifiable intervention strategies within this framework, allowing a structured assessment of bias across observational studies evaluating diet–disease relationships. Accordingly, the hypothetical target trial was conceptualized as comparing individuals exposed to different dietary patterns or nutrient intakes while aiming to emulate comparable baseline conditions, recognizing that dietary exposures were naturally occurring rather than assigned. Nevertheless, because these studies assessed naturally occurring dietary exposures rather than assigned interventions, ROBINS-I was applied pragmatically, and the resulting risk-of-bias assessments were interpreted cautiously, considering the methodological limitations inherent to observational exposure studies [34].

For the confounding domain, study-specific assessments were conducted by considering factors likely to influence both dietary exposure and the metabolic outcome under investigation. Rather than applying a uniform set of covariates across all studies, the evaluation was tailored to each study design and research question, considering demographic, anthropometric, metabolic, lifestyle and clinical characteristics, as well as population structure where appropriate. Investigators evaluated each study across the relevant bias domains and provided an overall risk of bias rating categorized as ‘low,’ ‘moderate, ’ ‘serious, ’ ‘critical, ’ or ‘unclear’ [35].

## 3. Results

The reporting of the available information is shown in the PRISMA checklist in Table S2 in Supporting Information online.

### 3.1. Search Procedure

**Figure 1:**
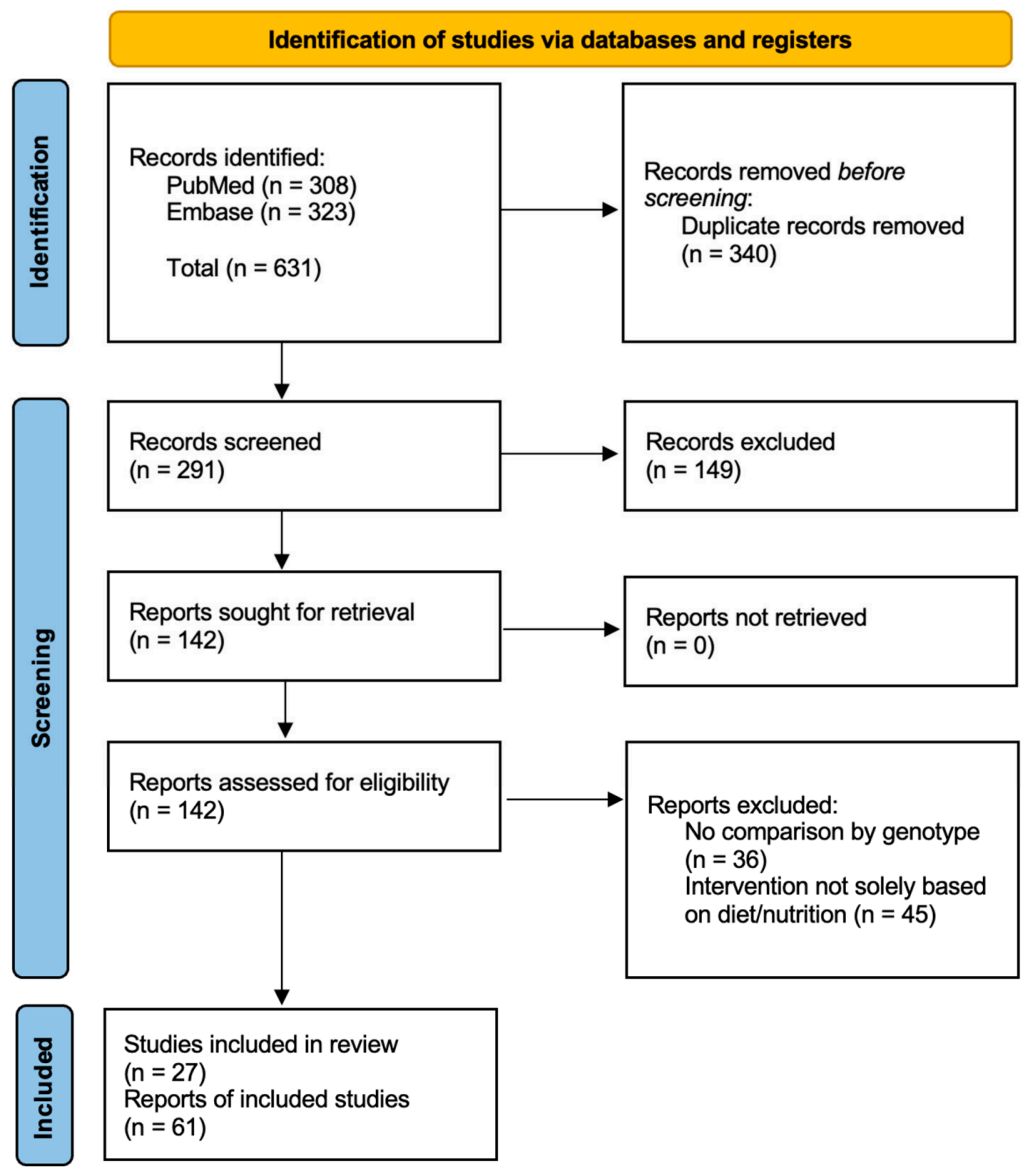
Flow diagram of the literature search process [31].

The search yielded 631 records. After removing duplicates (*n* = 340), 291 records were screened by title and abstract. Following screening, 142 full-text reports were assessed for eligibility. Ultimately, 61 reports representing 27 unique studies met the inclusion criteria. A detailed flow of the study selection process and reasons for exclusion is presented in Figure 1.

### 3.2. Characteristics of Included Studies

The characteristics of the included reports are presented in Table 2, Table 3 and Table 4. Among the 61 included reports, 23 were randomized controlled trials (RCTs) (37.7%), 7 were crossover RCTs (11.5%), 10 were cohort studies (16.5%), 8 were cross-sectional studies (13.1%), 8 were non-randomized dietary interventions (13.1%), 3 were case–control studies (4.9%), 1 was a case–cohort study (1.6%), and 1 was an exploratory nested case–control with confirmatory case–cohort design (1.6%).

Across these reports, 55 distinct SNPs in 40 genes were investigated. In addition, 11 reports used genetic risk scores (GRSs), incorporating between 6 and 116 SNPs or an unspecified number of diabetes-related variants, while one further report used an aggregate score based on two variants.

The genes most frequently examined included *TCF7L2*, *ADIPOQ*, *FTO*, *IRS1*, *GIPR*, *PPM1K*, *FABP2* and *BDNF*, although a wide range of loci involved in glucose metabolism, insulin signaling, lipid metabolism and energy regulation were represented. The included reports encompassed populations of predominantly European, Asian, and North American ancestry, although some studies were conducted in Middle Eastern and other populations. Dietary exposures included overall dietary patterns, macronutrient composition, dietary fat quality, specific foods, nutrient intakes, and controlled hypocaloric interventions. Although all included participants were free of diagnosed T2D at baseline, the study populations varied considerably with respect to metabolic characteristics, including metabolically healthy adults, overweight or obese individuals, and participants with cardiometabolic risk factors. Intervention duration and follow-up, where applicable, ranged from short-term dietary interventions lasting only a few weeks to prospective cohort studies with several years of follow-up.

### 3.3. Outcomes

Three main outcome categories were identified in the included studies: incident T2D risk (*n* = 13), insulin-related traits (*n* = 41), and glycemic traits and β-cell function (*n* = 7). Within each category, studies were further classified by type of dietary exposure. The findings of the included studies by outcome are shown in Table 2, Table 3 and Table 4.

Regarding Hardy–Weinberg equilibrium (HWE), 47 studies reported that genotype distributions were consistent with HWE, 1 study reported deviation from HWE, and 13 studies did not report HWE. HWE is commonly used in genetic studies to assess whether genotype frequencies in the study population are consistent with those expected in the source population [36].

### 3.4. Gene–Diet Interactions on Incident T2D/T2D Risk

#### 3.4.1. Polygenic Risk Scores and Overall Diet Quality/Dietary Patterns

Four studies examined whether overall diet quality or dietary patterns modified the association between GRS and T2D risk (Table 2A). In two large European cohort studies, no significant interaction was observed between GRS and adherence to either the EAT–Lancet diet index or the Mediterranean diet score in relation to incident T2D, although individuals with both high genetic risk and poorer diet quality had exhibited higher overall risk [37,38]. Similarly, Li et al. (2018) found no evidence that macronutrient composition modified associations between either a T2D GRS or an insulin-resistance GRS and incident T2D [39].

In contrast, Qi et al. (2009) reported a significant interaction between a 10-SNP GRS and a Western dietary pattern, such that higher adherence to the Western pattern was associated with greater T2D risk among individuals with high GRS, whereas no clear association was observed among lower-risk groups [40]. The same study also identified significant interactions for specific Western diet components, including red meat, processed meat, and heme iron intake [40].

**Table 2:** Gene–diet interactions on incident T2D/diabetes risk.

Study	Study Design	No. of Participants—All (M/F%)	Age (Years, Range/Mean)	Region, Ethnicity/Nationality	Gene(s)—SNP(s)	ΕAF	HWE Met	Intervention/Exposure	Outcome (Measure)	Result
A. Polygenic risk scores and overall diet quality/dietary patterns										
Langenberg et al., 2014 ([37])	Cohort	16,154 (49.8/50.2)	51.9 ± 9.5	European	49-SNP GRS	0.08–0.94	Y	Mediterranean diet score	Incident T2D	Formal interaction test: Not significant (all interaction *p* > 0.10). Main finding: No SNP–Mediterranean diet interaction remained significant after Bonferroni correction.
Zhang et al., 2023 ([38])	Cohort	23,441 (38.5/61.5)	58.1 ± 7.7	European	116-SNP GRS	NA	NA	EAT-Lancet diet index	Incident T2D: HRs; Population attributable risk (PAR) for highest diet-index category	Formal interaction test: Not significant (multiplicative *p* = 0.59; additive *p* = 0.44). Main finding: Participants with high genetic risk and low EAT-Lancet diet adherence had the highest T2D risk (HR 1.79, 95% CI 1.63–1.96).
Li et al., 2018 ([39])	Cohort	12,158 (37.9/62.1)	52.3 ± 9.3	European	GRS (number of SNPs NA)	NA	NA	Habitual dietary intake as % of total energy intake	T2D and insulin resistance incidence	Formal interaction test: Not significant (T2D GRS: Pint ≥ 0.20; insulin-resistance GRS: Pint ≥ 0.21). Main finding: Macronutrient composition did not modify the association between genetic risk and incident T2D.
Qi et al., 2009 ([40])	Case–Control	1337 (1337/0)	55.0 ± 9.0	European	10-SNP GRS	NA	Y	Prudent dietary pattern (vegetables, fruit, legumes, whole grains, fish, poultry) vs. Western dietary pattern (processed/red meat, butter, high-fat dairy, eggs, refined grains)	T2D risk	Formal interaction test: Significant (Western dietary pattern × GRS, Pinteraction = 0.02). Main finding: Greater adherence to the Western dietary pattern was associated with higher T2D risk only among individuals with high genetic risk (Q4 vs. Q1: OR 2.06, 95% CI 1.48–2.88; Ptrend = 0.01). Additional findings: The interaction remained significant after restricting the analysis to participants without prevalent T2D and after excluding smokers. Significant interactions were also observed for red meat, processed meat, and heme iron intake.
B. Genetic variants interacting with macronutrient intake										
Hong et al., 2018 ([41])	Cohort	8842 (53.8/46.2)	52.3 ± 8.9	Korea	6-SNP GRS	NA	NA	Habitual intake of 23 nutrients (Korean food composition table)	T2D (definition using fasting glucose/2 h OGTT glucose/antidiabetic medication); glucose intolerance; HbA1c; fasting insulin; HOMA-IR; HOMA-B.	Formal interaction test: Significant only for energy intake (Pinteraction = 0.0105). Main finding: Higher energy intake strengthened the association between the GRS and T2D risk. Compared with the medium genetic-risk group, the OR for T2D in the high genetic-risk group increased from 1.557 (95% CI 1.204–2.013) in the low-energy intake stratum to 2.080 (95% CI 1.416–3.058) in the high-energy intake stratum. Additional findings: No significant gene–diet interactions were observed for HOMA-B.
Kim et al., 2018 ([42])	Cohort	8842 (47.3/52.7)	40–69	Korea	10-SNP GRS	0.19–0.48	Y	Habitual energy, protein, carbohydrate, fat and calcium intake	T2D risk; HbA1c; HOMA-IR; HOMA-B	Formal interaction test: Not significant for carbohydrate intake (Pinteraction = 0.0727). No significant interactions were observed for HOMA-IR (*p* = 0.3888) or HOMA-B (*p* = 0.1122). Main finding: The association between the GRS and T2D risk appeared stronger among individuals with lower carbohydrate intake. In the low-carbohydrate group, the ORs for T2D were 1.91 (95% CI 1.52–2.40) for the medium GRS and 2.27 (95% CI 1.72–2.99) for the high GRS (vs. low GRS), compared with 1.94 (95% CI 1.18–3.18) for the high GRS in the high-carbohydrate group. Additional findings: Reduced HOMA-B with increasing genetic risk was observed primarily among individuals with lower carbohydrate intake.
Sonestedt et al., 2012 ([43])	Cohort	24,840 (38.6/61.4)	45–74	Caucasian	*GIPR* rs10423928	0.22	Y	macronutrient intake as % of non-alcohol energy	Incident T2D	Main finding: Higher carbohydrate intake reduced T2D risk among TT carriers by 23% (Ptrend = 0.005). (Ptrend = 0.005), whereas higher fat intake reduced T2D risk among AA carriers by 69% (HR = 0.31; 95% CI 0.14–0.71; P trend = 0.04). Additional findings: No significant interactions were observed for HbA1c or HOMA-IR. Formal interaction test: Significant for carbohydrate (Pinteraction = 0.0005) and fat intake (Pinteraction = 0.0006). Main finding: Higher carbohydrate intake was associated with a 23% lower risk of T2D among TT homozygotes (95% CI 5–38%; Ptrend = 0.005), whereas it was associated with an increased risk among AA homozygotes (Ptrend = 0.04). Conversely, among AA homozygotes, the highest quintile of fat intake was associated with a 69% lower risk of T2D (HR 0.31, 95% CI 0.14–0.71) compared with the lowest quintile.
Fisher et al., 2011 ([44])	nested case–control	2248 (38/62)	35–65	Germany	*CAV2* rs2270188	0.5	Y	total fat, SFA, MUFA, PUFA intake as % energy	Incident-T2D (HR/RR)	Formal interaction test: Significant for total fat (Pinteraction = 0.02) and saturated fat (Pinteraction = 0.002). Main finding: Higher total fat and saturated fat intake increased T2D risk among TT homozygotes (per +1% energy: fat RR = 1.06; *p* = 0.002 and SFA RR = 1.12; *p* = 0.0006).
Ericson et al., 2013 ([45])	Cohort	24,841 (38.7/61.3)	45–74	Sweden	*IRS1* rs2943641	0.37	Y	carbohydrate, protein, fat as % of energy and fiber	Incident T2D (mean follow-up 12 years); fasting glucose/insulin and HOMA-IR in 6103-person subsample	Formal interaction test: Significant for sex × genotype × carbohydrate (*p* = 0.01) and sex × genotype × fat (*p* = 0.01) interactions on incident T2D. Main finding: The protective association of the T allele with incident T2D differed according to sex and macronutrient intake. In women, the protective effect was observed only with low- and moderate-carbohydrate intake (both Ptrend = 0.01), with the lowest T2D risk among TT homozygotes consuming a low-carbohydrate diet (HR 0.51, 95% CI 0.32–0.81). In men, the protective effect was evident with low-fat intake (Ptrend = 0.01) and high-carbohydrate intake (Ptrend = 0.04), with the lowest T2D risk among TT homozygotes consuming a low-fat diet (HR 0.62, 95% CI 0.37–1.02).
C. Gene–food interactions										
Hindy et al., 2012 ([46])	Cohort	5216 (41/59)	M: 57.5 ± 6.0, F: 57.3 ± 5.9	Sweden	*TCF7L2* rs7903146	0.26	Y	Total energy (kJ); carbohydrate, fat, protein (% of non-alcohol energy); fiber (g per 4184 kJ/1000 kcal)	Incident T2D; baseline HbA1c; baseline fasting glucose	Formal interaction test: Significant for fiber intake (Pinteraction = 0.049). Main finding: Higher fiber intake was associated with a lower risk of incident T2D only among CC homozygotes (OR 0.74, 95% CI 0.58–0.94), whereas no protective association was observed among CT or TT carriers. Additional findings: The fiber × genotype interaction remained significant after excluding energy misreporters (*p* = 0.006) and dietary changers (*p* = 0.046). A significant interaction was also observed for baseline HbA1c (Pinteraction = 0.020), with the T allele associated with higher HbA1c only in the highest fiber quintile (Ptrend = 0.002), whereas no interaction was observed for fasting glucose.
Fisher et al., 2009 ([47])	Case–Cohort	2318 (NA)	35–65	Germany	*TCF7L2* rs7903146	0.25	NA	Whole-grain intake modeled per 50 g/day portion	Incident T2D (weighted Cox; HR)	Formal interaction test: Significant (Pinteraction = 0.016). Main finding: Higher whole-grain intake was associated with lower T2D risk among C homozygotes (HR per 50 g/d = 0.86, 95% CI 0.75–0.99) but not among T-allele carriers (HR per 50 g/d = 1.08, 95% CI 0.96–1.23).
Corella et al., 2016 ([48])	RCT	7098 (42.7/57.3)	M: 55–80/F: 60–80	Caucasian	*CLOCK* rs4580704	0.38	Y	Mediterranean diet (MedDiet) + extra-virgin olive oil (EVOO) or MedDiet + nuts vs. low-fat control diet	Fasting glucose (baseline, non-T2D); incident T2D	Formal interaction test: Not significant (Pinteraction = 0.052). Main finding: G-allele carriers had a lower risk of incident T2D than CC homozygotes (HR = 0.68, 95% CI 0.54–0.86; multivariable HR = 0.69, 95% CI 0.54–0.87). The protective association was more pronounced in the Mediterranean diet intervention groups (HR = 0.58, 95% CI 0.43–0.78) than in the low-fat control group (HR 0.95, 95% CI 0.63–1.44).
Lamri et al., 2016 ([49])	Cohort	5212 (NA)	30–65	Caucasian	*FFAR4* rs116454156	0.01	NA	Dietary fat intake as g/day and % energy	Incident T2D (ADA: fasting plasma glucose ≥ 7.0 mmol/L or diabetes treatment) over 9 years	Formal interaction test: Significant for dietary fat expressed as g/day (Pinteraction = 0.04) and sex-specific tertiles (Pinteraction = 0.03); not significant when fat was expressed as percentage of energy. Main finding: Among low-fat consumers, RH carriers had a higher risk of incident T2D than RR homozygotes (OR 4.81 [1.52–13.64], *p* = 0.004).

ADA, American Diabetes Association; CHO, carbohydrate; CI, confidence interval; EAF, effect allele frequency; EVOO, extra-virgin olive oil; HR, hazard ratio; kcal, kilocalories; kJ, kilojoules; MedDiet, Mediterranean diet; M/F, male/female; MUFA, monounsaturated fatty acids; NA, not available/not reported; OR, odds ratio; PAR, population attributable risk; PUFA, polyunsaturated fatty acids; Q, quartile; RCT, randomized controlled trial; RH, Arg/His heterozygote; RR, Arg/Arg homozygote; R270H, Arg270His variant; RR, relative risk; SFA, saturated fatty acids; %E, percentage of total energy intake.

**Table 3:** Gene–diet interactions for insulin resistance/insulin sensitivity/insulin-related traits.

Study	Study Design	No. of Participants—All (M/F%)	Age (Years, Range/Mean)	Region, Ethnicity/Nationality	Gene(s)—SNP(s)	EAF	HWE Met	Intervention/ Exposure	Outcome (Measure)	Result
A. Macronutrient composition in energy-restricted diets										
de Luis et al., 2017a [50]	RCT	361 (28/72)	44.8 ± 9.9	Caucasian	*BDNF* rs10767664	0.16	Y	Hypocaloric diet enriched in PUFA (sunflower oil 30 mL/day + fish + nuts; ~1448 kcal/day) vs. hypocaloric diet enriched in MUFA (virgin olive oil 30 mL/day; ~1442 kcal/day); macronutrient distributions similar.	Plasma glucose; insulin; HOMA-IR	Formal interaction test: Significant genotype–diet interaction for insulin (Pinteraction = 0.01) and HOMA-IR (Pinteraction = 0.02). Main finding: Following the MUFA-enriched hypocaloric diet, non-T allele carriers showed greater reductions in insulin (−2.1 ± 2.0 vs. −0.7 ± 2.9 IU/L; *p* = 0.009) and HOMA-IR (−0.7 ± 0.9 vs. −0.3 ± 1.0; *p* = 0.008) than T-allele carriers.
Huang et al., 2015 ([51])	RCT	730 (39/61)	30–70	Mixed	*PCSK7* rs236918	0.12	NA	low- vs. high-carbohydrate weight-loss diets (dietary carbohydrate level used as effect modifier; adherence supported by biomarkers) for 2 years	Fasting insulin; HOMA-IR	Formal interaction test: Significant genotype–carbohydrate interaction for fasting insulin during the weight-loss phase (Pinteraction = 0.04), with stronger interactions in adjusted analyses for fasting insulin and HOMA-IR (Pinteraction = 0.001 for both). Main finding: During the first 6 months, the G allele was associated with greater reductions in fasting insulin and HOMA-IR in participants assigned to high-carbohydrate diets, whereas opposite genotype effects were observed in low-carbohydrate diets. Additional findings: Gene–diet interactions were attenuated during the weight-regain phase but remained evident for HOMA-IR in the overall cohort (Pinteraction = 0.034), with stronger interactions when the most extreme carbohydrate diets were compared.
Goni et al., 2017 ([52])	RCT	757 (25/75)	20–50	Caucasian	*PPM1K* rs1440581	0.51	Y	Low-fat/high-carbohydrate vs. high-fat/low-carbohydrate weight-loss diets for 10 weeks	Fasting glucose; fasting insulin; HOMA-IR; HOMA-B	Formal interaction test: Significant genotype–diet interactions for insulin after adjustment for weight loss (Pinteraction = 0.006), HOMA-IR (Pinteraction = 0.01), and HOMA-B (Pinteraction = 0.002). Main finding: In the low-fat/high-carbohydrate group, each T allele was associated with smaller improvement in insulin markers (Δinsulin β = +0.69 µU/mL, *p* = 0.07; ΔHOMA-IR β = +0.21, *p* = 0.04). In the high-fat/low-carbohydrate group, the T allele was associated with greater improvement (e.g., Δinsulin β = −0.64, *p* = 0.09; and ΔHOMA-B decreased by 12.5 units per T allele, *p* = 0.006).
Han et al., 2017 ([53])	RCT	680 (39.6/60.4)	30–70	Mixed (white and black)	8-SNP GRS	NA	NA	2 × 2 factorial diets: low-fat vs. high-fat × average-protein vs. high-protein	Fasting glucose; fasting insulin; HOMA-IR	Formal interaction test: Significant GRS × dietary fat interactions for fasting insulin (Pinteraction = 0.023) and HOMA-IR (Pinteraction = 0.022) at 6 months. Main finding: At 6 months, participants assigned to the low-fat diet exhibited progressively greater reductions in fasting insulin and HOMA-IR across increasing GRS tertiles, whereas no significant association between GRS and changes in these glycaemic traits was observed in the high-fat diet group. Additional findings: No significant GRS × dietary fat interactions were observed after 2 years.
Schüler et al., 2017 ([54])	Non-randomized intervention	92 (37/63)	31.0 ± 14.0	Caucasian	*ACE* rs4343	0.42	Y	Sequential isocaloric diet: 6-wk carbohydrate-rich low-fat (LF: 55% carbohydrate, 30% fat, 15% protein) → 6-wk high-fat (HF: 40% carbohydrate, 45% fat, 15% protein; emphasis on saturated fat).	Fasting glucose, fasting insulin, HOMA-IR; ivGTT-derived glucose/insulin responses (iAUC glucose, iAUC insulin, 2h glucose; AIRg	Formal interaction test: Significant genotype × high-fat diet interactions for fasting glucose (Pinteraction = 0.001), HOMA-IR (Pinteraction = 0.008), and ivGTT-derived glucose response (iAUC glucose; Pinteraction = 0.014). Main finding: During the HF diet, GG increased fasting glucose by +0.5 ± 0.1 mmol/L (*p* = 0.009) vs. no change in AA/AG (*p* = 0.191). HOMA-IR increased in GG (*p* = 0.002) but not AA/AG (*p* = 0.075); GG was higher than AA/AG at completion (*p* = 0.022). iAUC glucose increasing in GG only (*p* = 0.001) and ∆iAUC glucose differing by genotype (*p* = 0.009); iAUC insulin higher in GG at completion (*p* = 0.027); 2h glucose higher in GG at HF (*p* = 0.003). Fasting insulin increased in both genotypes (*p* = 0.038 AA/AG; *p* = 0.039 GG), but was higher in GG vs. AA/AG at completion (*p* = 0.042).
de Luis et al., 2016 ([55])	RCT	283 (26.5/73.5)	C/C 52.9 ± 11.2; T-carriers 52.3 ± 10.4	Caucasian	*UCP3* rs1800849	0.15	Y	Diet HP (1050 kcal/day; 33% carbohydrate, 33% fat, 34% protein) vs. Diet S (1093 kcal/day; 53% carbohydrate, 27% fat, 20% protein), 9 months	Fasting glucose, insulin, HOMA-IR	Formal interaction test: No formal genotype–diet interaction test reported. Main finding: Only CC carriers experienced significant reductions in fasting glucose (−5.2 ± 2.2 mg/dL), insulin (−3.9 ± 3.1 mIU/L), and HOMA-IR (−0.6 ± 0.1) (all *p* < 0.05) following the HP diet, whereas T-allele carriers showed no significant improvements.
de Luis et al., 2018 [56]	RCT	284 (26.8/73.2)	48.2 ± 10.9	Caucasian	*ADIPOQ* rs1501299	0.29	Y	Diet I: hypocaloric moderate-carbohydrate (1507 kcal/day; 38% carbohydrate, 26% protein, 36% fat) vs. Diet II: hypocaloric low-fat (1500 kcal/day; 53% carbohydrate, 20% protein, 27% fat), 12 weeks	Fasting glucose, fasting insulin, HOMA-IR	Formal interaction test: No formal genotype–diet interaction test reported. Main finding: GG homozygotes showed significant reductions in fasting insulin and HOMA-IR after weight loss with both hypocaloric diets, with no difference in the magnitude of improvement between dietary patterns (Insulin: Diet I −4.7 ± 1.4 vs. Diet II −5.9 ± 1.9 mIU/L; *p* = 0.76. HOMA-IR: Diet I −1.4 ± 0.6 vs. Diet II −2.0 ± 0.7; *p* = 0.56).
Huang et al., 2016 ([57])	RCT	744 (NA)	30–70	Mixed (white and black)	31-SNP GRS	NA	NA	2 × 2 factorial weight-loss diets varying protein (15% vs. 25% energy) and fat (20% vs. 40% energy) over 2 years (carbohydrate accordingly 35–65% energy)	Fasting insulin; HOMA-IR; HOMA-B; HbA1c	Formal interaction test: Significant genotype–diet interaction between dietary protein and GRS for fasting insulin (Pinteraction = 0.02), HOMA-IR (Pinteraction = 0.05), HOMA-B (Pinteraction = 0.01), and HbA1c (Pinteraction = 0.04) among White participants. Main finding: Lower GRS was associated with greater improvements in fasting insulin, HbA1c, HOMA-IR and HOMA-B (Ptrend= 0.04, 0.0001, 0.02 and 0.004) in participants consuming the low-protein diet. In the high-protein group, the insulin pattern was opposite (Ptrend = 0.03).
de Luis et al., 2020 ([58])	RCT	270 (NA)	49.4 ± 6.2	Caucasian	*MTNR1B* rs10830963	0.27	Y	Diet HP (high-protein/low-carbohydrate severe hypocaloric): ~1023.9 kcal/d; 33.5% carbohydrate, 32.5% fat, 34.5% protein vs. Diet S (standard protein severe hypocaloric): ~1028.7 kcal/d; 52.9% carbohydrate, 27.2% fat, 19.9% protein, 9 months	Fasting glucose, fasting insulin, HOMA-IR	Formal interaction test: No formal genotype-diet interaction test reported. Main finding: after 9 months, CC homozygotes showed greater reductions in fasting insulin and HOMA-IR than CG/GG carriers following both the HP (insulin: Δ −4.7 ± 0.8 vs. −0.9 ± 1.0; *p* = 0.03; HOMA-IR: Δ −0.8 ± 0.2 vs. −0.2 ± 0.1; *p* = 0.03) and S hypocaloric diets (insulin: Δ −3.4 ± 0.6 vs. −1.2 ± 0.4; *p* = 0.02 and HOMA-IR: Δ −1.1 ± 0.2 vs. −0.1 ± 0.1; *p* = 0.01).
Qi et al., 2015 ([59])	RCT	732 (38.8/61.2)	30–70	Mixed (white and black)	*DHCR7* rs12785878; *CYP2R1* rs10741657; GC rs2282679	rs12785878 (T) = 0.67; rs10741657 (A) = 0.36; rs2282679 (C) = 0.24	Y	Energy-reduced diets (−750 kcal/day); 2 × 2 factorial: low-fat (20%) vs. high-fat (40%) and low-protein (15%) vs. high-protein (25%) for 2 years	Fasting insulin, fasting glucose, HOMA-IR	Formal interaction test: Significant protein × DHCR7 rs12785878 interaction for fasting insulin (Pinteraction = 0.0008) and HOMA-IR (Pinteraction = 0.0009) at 6 months. Main finding: In the high-protein diet group, each T allele of DHCR7 rs12785878 was associated with greater reductions in fasting insulin (β = −0.13 ± 0.04; *p* = 0.0009) and HOMA-IR (β = −0.13 ± 0.04; *p* = 0.002), whereas no genotype effect was observed in the low-protein diet group. Additional findings: The associations were attenuated after 2 years. No significant gene–diet interactions were observed for CYP2R1 or GC variants after correction for multiple testing.
Xu et al., 2013 ([60])	RCT	734 (39.0/61.0)	30–70	Mixed	*PPM1K* rs1440581	0.55	Y	Energy-reduced diets; low-fat (20%) vs. high-fat (40%) (2 × 2 factorial also varying protein 15% vs. 25%), 2 years	Insulin resistance: fasting insulin (µU/mL; log-change) and HOMA-IR (log-change)	Formal interaction test: Significant genotype–fat interaction for fasting insulin (Pinteraction = 0.006) and HOMA-IR (Pinteraction = 0.006) at 6 months. Main finding: At 6 months, CC homozygotes assigned to the low-fat diet exhibited greater reductions in fasting insulin (log-change: −0.33 vs. −0.27 and −0.14 for CT and TT, respectively) and HOMA-IR (−0.36 vs. −0.29 and −0.18), whereas TT homozygotes assigned to the high-fat diet showed the greatest improvements (fasting insulin: −0.34 vs. −0.30 and −0.16; HOMA-IR: −0.38 vs. −0.32 and −0.17 for TT, CT and CC, respectively). Additional findings: No significant genotype × dietary fat interactions were observed after 2 years (Pinteraction ≥ 0.13).
Qi et al., 2012 ([61])	RCT	737 (39.0/61.0)	51.0 ± 9.0	Mixed	*GIPR* rs2287019	0.17	Y	Energy-reduced diets; low-fat (20%) vs. high-fat (40%) (2 × 2 factorial also varying protein 15% vs. 25%), 2 years	% change from baseline in fasting glucose, fasting insulin, HOMA-IR	Formal interaction test: A significant genotype × dietary fat interaction was observed for fasting glucose (Pinteraction = 0.04), whereas the interactions for fasting insulin (Pinteraction = 0.10) and HOMA-IR (Pinteraction = 0.07) were not statistically significant. Main finding: At 6 months, among participants assigned to the low-fat diet, T-allele carriers showed greater reductions in fasting glucose (β = −2.33 ± 0.86%; *p* = 0.006), fasting insulin (β = −8.76 ± 4.13%; *p* = 0.03), and HOMA-IR (β = −10.52 ± 4.39%; *p* = 0.01), whereas no genotype effect was observed in the high-fat diet. Additional findings: The genotype-specific improvements observed at 6 months were attenuated after 2 years.
Grau et al., 2010 ([62])	RCT	739 (24.9/75.1)	20–50	European	*TCF7L2* rs7903146	0.28	Y	Hypoenergetic diet (~2600 kcal/d) randomized to HF (40–45% energy from fat; low-carbohydrate) vs. LF (20–25% energy from fat; high-carbohydrate), 10 weeks	ΔHOMA-IR	Formal interaction test: Significant genotype–diet interaction (Pinteraction = 0.0025). Main finding: Among TT homozygotes, the LF diet produced a greater reduction in HOMA-IR than the high-fat diet (difference = 1.33 units; *p* = 0.004).
Grau et al., 2009 ([63])	RCT	771 (24.9/75.1)	20–50	European	*FTO* rs9939609	0.42	Y	Two hypo-caloric diets (~600 kcal/day deficit): LF (20–25%E fat; 60–65%E carbohydrate) vs. HF (40–45%E fat; 40–45%E carbohydrate)	ΔHOMA-b, ΔHOMA-IR,	Formal interaction test: Significant genotype × dietary fat interactions were observed for HOMA-β (Pinteraction = 0.0083) and HOMA-IR (Pinteraction = 0.047). Main finding: TT homozygotes exhibited greater improvements in β-cell function following the low-fat diet than the high-fat diet, with a 19-unit greater reduction in HOMA-β (*p* = 0.006). Compared with AT/AA carriers on the low-fat diet, TT homozygotes also showed an 18-unit greater reduction in HOMA-β (*p* = 0.004). For HOMA-IR, TT homozygotes assigned to the low-fat diet showed a 0.44-unit greater reduction than AT/AA carriers (*p* = 0.013), whereas the difference between low-fat and high-fat diets within TT homozygotes did not reach statistical significance (0.30 units; *p* = 0.14).
Zheng et al., 2015 ([64])	RCT	743 (38.8/61.2)	30–70	Mixed (white and black)	*FTO* rs1558902; *FTO* rs9939609	rs1558902 A = 0.40; rs9939609 A = 0.45	Y	Four energy-limited diets (750 kcal/d deficit): fat 20% vs. 40% × protein 15% vs. 25% (carbohydrate accordingly)	Fasting glucose, Fasting insulin, HOMA-IR, HOMA-b	Formal interaction test: Significant genotype × dietary fat interactions were observed for FTO rs1558902 on fasting insulin (Pinteraction = 0.004) and HOMA-IR (Pinteraction = 0.003). No significant genotype–diet interactions were observed for FTO rs9939609. Main finding: At 6 months, the effect of the rs1558902 A allele differed according to dietary fat intake. In the low-fat diet group, each A allele was associated with smaller reductions in fasting insulin (β = +0.05 for Δlog insulin; *p* = 0.06) and HOMA-IR (β = +0.05 for Δlog HOMA-IR; *p* = 0.06), whereas no significant genotype effects were observed in the high-fat diet group (β = −0.04 for both outcomes; *p* > 0.1).
Wang et al., 2016 ([65])	RCT	733 (39.2/60.8)	30–70	Mixed	14-SNP GRS	0.02–0.71	Y	Four energy-reduced diets (750 kcal/d deficit): 20/15/65; 20/25/55; 40/15/45; 40/25/35 (%fat/%protein/–carbohydrate). Analyses combined into low-fat (20%) vs. high-fat (40%) groups.	Fasting glucose, fasting insulin, HOMA-IR, HOMA-S (changes at 6 months and 2 years)	Formal interaction test: Significant genotype × dietary fat interactions were observed for fasting glucose (Pinteraction = 0.007), HOMA-IR (Pinteraction = 0.045), and HOMA-S (Pinteraction = 0.028) at 6 months. Main finding: At 6 months, a higher GRS was associated with less favorable glycaemic changes, including fasting glucose (*p* < 0.001), fasting insulin (*p* = 0.042), HOMA-IR (*p* = 0.009), and HOMA-S (*p* = 0.043). In the high-fat dietgroup, fasting glucose increased progressively across GRS tertiles (T1: −0.07 ± 0.09, T2: 0.02 ± 0.09, T3: 0.14 ± 0.09 mmol/L; Ptrend < 0.001), whereas no significant trend was observed in the low-fat diet group (Ptrend = 0.087). Additional findings: After further adjustment for weight loss, the genotype × dietary fat interaction remained significant for fasting glucose (*p* = 0.015). All GRS associations and genotype–diet interactions were attenuated after 2 years.
Qi et al., 2011 ([66])	RCT	738 (39.0/61.0)	51.0 ± 9.0	Mixed	*IRS1* rs2943641	0.34	Y	Energy-reduced diets randomized to four macronutrient targets (%fat/%protein/%carbohydrate): 20/15/65; 20/25/55; 40/15/45; 40/25/35 (carbohydrate-rich foods lower GI)	Glucose, Insulin, HOMA-IR	Formal interaction test: Significant genotype × dietary carbohydrate interaction for changes in log fasting insulin (Pinteraction = 0.024) and log HOMA-IR (Pinteraction = 0.025) at 6 months. Main finding: At 6 months, CC homozygotes assigned to the highest-carbohydrate/low-fat diet (65% carbohydrate, 20% fat) exhibited greater reductions in log fasting insulin (*p* = 0.006) and log HOMA-IR (*p* = 0.009) than CT/TT carriers. Conversely, in the lowest-carbohydrate diet (35% carbohydrate), CC homozygotes showed smaller reductions in these markers than CT/TT carriers (borderline significance). Additional findings: At 2 years, the genotype–diet interactions were attenuated after correction for multiple testing. In secondary analyses comparing the most extreme diets, CC homozygotes benefited more from the high-carbohydrate than the low-carbohydrate diet (fasting insulin *p* = 0.016; HOMA-IR *p* = 0.009), whereas the opposite pattern was observed among non-CC carriers (fasting insulin *p* = 0.03; HOMA-IR *p* = 0.05).
Huang et al., 2018 ([67])	RCT	POUNDS Lost: 722 (37.1/62.9); DIRECT 170 (87.1/12.9)	30–70	USA (POUNDS Lost); Israel (DIRECT)	*HNF1A* rs7957197	0.05	Y	Hypocaloric diets varying macronutrients; primary contrast low-fat (20%) vs. high-fat (40%/low-carbohydrate); protein contrasts also tested.	Insulin, HOMA-IR	Formal interaction test: Significant genotype × dietary fat interactions were observed for fasting insulin (Pinteraction = 0.02) and HOMA-IR (Pinteraction = 0.03) at 6 months. Main finding: In the pooled analysis, T-allele carriers assigned to the high-fat diet exhibited greater reductions in fasting insulin (*p* = 0.03) and HOMA-IR (*p* = 0.01) than non-carriers. In contrast, no significant genotype differences were observed among participants assigned to the low-fat diet (fasting insulin: *p* = 0.81; HOMA-IR: *p* = 0.67). Additional findings: No significant genotype × dietary fat interactions were observed after 2 years in either trial.
B. Fat quality and Mediterranean-type diets										
Moreno et al., 2005 ([68])	RCT	43 (51.2/48.8)	23.1 ± 1.8	Spain	*APOE* rs405509	0.52	NA	Three controlled diet periods (4 weeks each): SFA-rich (38% fat; 20% SFA) → then randomized crossover to MUFA-rich (38% fat; 22% MUFA) and carbohydrate-rich (30% fat; 55% carbohydrate); cholesterol ~300 mg/d	Steady-state plasma glucose (SSPG; insulin suppression test; insulin sensitivity proxy)	Formal interaction test: A significant diet × genotype interaction was reported for SSPG, and fasting insulin concentrations (exact Pinteraction not reported). Main finding: Switching from the SFA-rich diet to either the MUFA-rich or high-carbohydrate diet reduced SSPG in GG and GT carriers (*p* < 0.05), but not in TT homozygotes, indicating greater improvement in insulin sensitivity among carriers of the −219G allele. Overall SSPG concentrations were lowest in GG homozygotes (4.85 ± 0.86 mmol/L), followed by GT (6.43 ± 0.48 mmol/L) and TT homozygotes (7.30 ± 0.77 mmol/L; *p* = 0.045).
Marín et al., 2005 ([69])	Crossover RCT	59 (NA)	21.0 ± 2.0	Spain	*FABP2* rs1799883	0.29	NA	Three diets, 4 weeks each: SFA-rich (38% fat; 20% SFA) → then randomized crossover to carbohydrate (28% fat; <10% SFA) and Mediterranean/MUFA (38% fat; 22% MUFA)	Steady-state plasma glucose (SSPG; insulin suppression test; insulin sensitivity proxy)	Formal interaction test: Borderline significant genotype × diet interaction for SSPG (Pinteraction = 0.050). Main finding: Thr54 carriers exhibited improved insulin sensitivity following the MUFA-rich and high-carbohydrate diets compared with the SFA-rich diet, with SSPG decreasing from 7.8 ± 4.2 mmol/L after the SFA-rich diet to 6.7 ± 3.1 mmol/L after the high-carbohydrate diet and 6.1 ± 2.5 mmol/L after the MUFA-rich diet (*p* < 0.05 for both comparisons). In contrast, Ala54 homozygotes showed no significant differences in SSPG across diets (6.4 ± 2.8, 5.6 ± 2.4, and 5.5 ± 2.8 mmol/L, respectively).
de Luis et al., 2019 [70]	RCT	363 (28.1/71.9)	49.7 ± 7.9	Caucasian	*ADIPOQ* rs1501299	0.73	Y	Diet M (enriched MUFA hypocaloric) vs. Diet P (enriched PUFA hypocaloric), 12 weeks	Insulin (mU/L), HOMA-IR	Formal interaction test: Not reported. Main finding: GG homozygotes showed greater improvements in insulin resistance than GT/TT carriers following both hypocaloric diets. After the P diet, fasting insulin decreased by −3.2 ± 1.0 vs. −0.6 ± 0.4 mU/L (*p* = 0.01) and HOMA-IR by −1.1 ± 0.2 vs. −0.3 ± 0.4 (*p* = 0.02). After the M diet, fasting insulin decreased by −3.7 ± 0.9 vs. −0.4 ± 0.5 mU/L (*p* = 0.01) and HOMA-IR by −1.0 ± 0.2 vs. −0.4 ± 0.3 (*p* = 0.03).
Dworatzek et al., 2004 ([71])	Crossover RCT	52 (46/54)	30.2 ± 2.0	Canada	*FABP2* rs1799883	0.22	Y	safflower oil vs. olive oil vs. butter	postprandial glucose, insulin, C-peptide (including incremental AUC), insulin:C-peptide ratio	Formal interaction test: Not reported. Main finding: Thr54 carriers exhibited lower postprandial insulin responses than Ala54 homozygotes across all dietary interventions, with insulin AUC (0–3 h) of 293 ± 52 vs. 384 ± 37 pmol·h/L (safflower oil), 322 ± 54 vs. 454 ± 63 pmol·h/L (olive oil), and 320 ± 51 vs. 436 ± 62 pmol·h/L (butter) (genotype main effect *p* = 0.05).
Pérez-Martínez et al., 2008 ([72])	Crossover RCT	59 (50.8/49.2)	23.1 ± 1.8	Spain	*ADIPOQ* rs17300539; rs266729; rs2241766; rs1501299	rs266729 (G) = 0.25; rs17300539 (A) = 0.16; rs2241766 (G) = 0.14; rs1501299 (T) = 0.27	Y	3 × 4-week diets: SFA-rich (baseline) → MUFA-rich and carbohydrate-rich in randomized crossover (SFA: 38% fat/20% SFA; MUFA: 38% fat/22% MUFA; carbohydrate: ~30% fat/55% carbohydrate)	Insulin resistance (SSPG from insulin suppression test; mmol/L)	Formal interaction test: Significant sex × genotype × diet interaction for ADIPOQ rs266729 (*p* = 0.016). Main finding: Among men only, CC homozygotes exhibited greater improvements in insulin sensitivity after switching from the SFA-rich diet to the MUFA-rich (SSPG: 8.95 ± 0.60 → 6.04 ± 0.31 mmol/L) or high-carbohydrate diet (8.95 ± 0.60 → 6.35 ± 0.38 mmol/L) than G-allele carriers (MUFA: 6.65 ± 0.40 → 6.45 ± 0.40; carbohydrate: 6.65 ± 0.40 → 5.83 ± 0.30 mmol/L).
Luan et al., 2001 ([73])	Cohort	592 (43.8/56.2)	53.9 ± 10.9	Caucasian	*PPARγ* rs1801282	0.12	Y	Dietary P:S ratio	fasting insulin	Formal interaction test: Significant genotype × dietary polyunsaturated-to-saturated fat (P:S) ratio interaction for fasting insulin (Pinteraction = 0.0097). Main finding: As the dietary P:S ratio increased, fasting insulin concentrations progressively decreased in Ala12 carriers, whereas no corresponding trend was observed in Pro12 homozygotes, consistent with a significant genotype × dietary fat interaction (interaction coefficient β = 0.548; *p* = 0.0097). Additional findings: The interaction was attenuated after adjustment for BMI (*p* = 0.18), suggesting that the association was partly mediated by adiposity.
de Luis et al., 2013 ([74])	Non-randomized intervention	126 (23/77)	48.3 ± 14.2	Spain	*LEPR* rs1805094	0.18	Y	Three-month hypocaloric high-MUFA diet (~1342 kcal; 46.6% carbohydrate, 34.1% fat, 19.2% protein; fat: 67.5% MUFA, 21.7% SFA, 10.8% PUFA)	insulin, HOMA-IR	Formal interaction test: Not reported. Main finding: Following the high-MUFA hypocaloric diet, Lys/Lys homozygotes showed significant reductions in fasting insulin (13.6 ± 8.5 → 12.1 ± 9.1 mU/L; Δ −1.5 ± 4.6, *p* < 0.05) and HOMA-IR (3.4 ± 2.4 → 3.0 ± 2.4; Δ −0.4 ± 1.9, *p* < 0.05), whereas Asn carriers showed no significant changes. Additional findings: Between-genotype differences in response were not formally tested.
Pérez-Martínez et al., 2007 ([75])	Crossover RCT	59 (50.8/49.2)	23.1 ± 1.8	Spain	*APOB* rs934197	0.19	Y	3 × 4-week diets: SFA-rich (38% fat, 20% SFA; baseline) then MUFA-rich (38% fat, 22% MUFA) and carbohydrate-rich (~30% fat, 55% carbohydrate) in randomized crossover	Insulin resistance (SSPG from insulin suppression test; mmol/L)	Formal interaction test: Significant sex-specific genotype × diet interaction in men (*p* = 0.040). Main finding: Male T-allele carriers exhibited greater improvements in insulin sensitivity after switching from the SFA-rich diet to either the MUFA-rich or high-carbohydrate diet than CC homozygotes. SSPG decreased from 9.18 ± 1.35 mmol/L after the SFA-rich diet to 6.55 ± 0.74 mmol/L after the MUFA-rich diet and 6.31 ± 0.93 mmol/L after the high-carbohydrate diet. No genotype effect was observed in women (*p* = 0.391).
Pérez-Martínez et al., 2005 ([76])	Crossover RCT	59 (50.8/49.2)	23.1 ± 1.8	Spain	*SCARB1* rs4238001	0.14	Y	28 d SFA-enriched run-in, then randomized crossover: 28 d MUFA-enriched (olive oil) and 28 d high-carbohydrate/low-fat diet (sequence randomized)	Insulin sensitivity (SSPG during insulin suppression test; lower = higher sensitivity)	Formal interaction test: Not reported. Main finding: After the MUFA-rich diet, G/A carriers exhibited lower SSPG than G/G homozygotes (4.41 ± 1.59 vs. 6.16 ± 2.78 mmol/L; *p* = 0.030), indicating greater insulin sensitivity. No genotype differences were observed after the SFA-rich diet (*p* = 0.957), and only a non-significant trend was observed after the high-carbohydrate diet (*p* = 0.177).
Gómez et al., 2005 ([77])	Crossover RCT	59 (50.8/49.2)	22.6 ± 1.4	Spain	*LIPC* rs1800588	NA	NA	Eight d SFA-enriched run-in, then randomized crossover: 28 d high-MUFA Mediterranean (olive oil-enriched) and 28 d low-fat high-carbohydrate	Insulin sensitivity (SSPG); fasting FFA/NEFA	Formal interaction test: Significant diet × genotype interaction in men (Pinteraction = 0.042). Main finding: Among men, −514T allele carriers (CT/TT) exhibited significantly higher SSPG (lower insulin sensitivity) after the SFA-rich diet than after both the high-carbohydrate and Mediterranean diets (*p* < 0.05 for both comparisons), whereas CC homozygotes showed no significant differences in SSPG across dietary interventions. No significant diet, genotype, or diet × genotype effects were observed in women.
Marín et al., 2011 ([78])	RCT	59 (50.8/49.2)	23.1 ± 1.8	Spain	*IRS1* rs1801278	0.05	Y	28 d SFA-enriched diet (run-in), then randomized crossover 28 days high-carbohydrate/low-fat diet and 28 days high-MUFA Mediterranean (olive oil) diet (cholesterol constant)	Fasting glucose, Insulin sensitivity (SSPG)	Formal interaction test: Significant genotype × diet interaction for SSPG (Pinteraction < 0.001). Main finding: Following the high-carbohydrate diet, G972R (GR) carriers exhibited significantly lower SSPG than GG homozygotes (*p* < 0.05), whereas no genotype differences were observed after the SFA-rich or MUFA-rich diets. Additional findings: Overall, SSPG was lower following both the MUFA-rich (6.2 ± 3.1) and high-carbohydrate (6.6 ± 3.1) diets than after the SFA-rich diet (8.1 ± 4.1), irrespective of genotype.
C. Other dietary exposures										
Holzbach et al., 2022 ([79])	Cross-Sectional	116 (27.6/72.4)	22.5 ± 3.5	Brazil	*PLIN1* rs894160	0.25	Y	Dietary intake reported as % of total energy intake	HOMA-IR; HbA1c; insulin	Formal interaction test: Significant carbohydrate × genotype interaction for HOMA-IR (Pinteraction = 0.027), remaining significant after multivariable adjustment (Pinteraction < 0.001). Main finding: Dietary carbohydrate intake modified the association between PLIN1 rs894160 and HOMA-IR. In the unfolded model, GA/AA carriers showed a marginally higher HOMA-IR than GG homozygotes (β = 0.032, *p* = 0.053), whereas the carbohydrate × genotype interaction remained significant after adjustment.
Lewis et al., 2023 ([80])	Crossover RCT	63 (46/54)	56.0 ± 10.0	Greenland Inuit	*TBC1D4* rs61736969	0.17	N	Two 4-week diet periods: Traditional Inuit diet (high fat >40%E; low carbohydrate <30%E; marine mammals/fish/reindeer; minimal grains/imported foods) vs. Western diet (high carbohydrate 55–65%E; moderate fat 30–35%E; lower marine fats, higher saturated fats from imported meats).	FPG; OGTT glucose (30 min, 120 min); IFG/IGT/diabetes (WHO); HbA1c; CGM metrics (24 h mean/min/max/range); insulin resistance/sensitivity indices (HOMA-IR, ISI_0–120_, BIGTT-Si, BIGTT-AIR).	Formal interaction test: A diet × genotype interaction was observed for ISI_0–120_ (exact Pinteraction not reported). No significant genotype × diet interactions were observed for HOMA-IR, BIGTT-Si, BIGTT-AIR, fasting glucose, fasting insulin, 2 h insulin, or HbA1c (*p* = 0.368). Main finding: Following the Traditional Inuit diet, TBC1D4 variant carriers had lower ISI_0–120_ than non-carriers (1.0 [IQR 1.0–1.2] vs. 1.8 [1.5–2.3]). After the Western diet, this difference was attenuated (1.3 [1.0–1.5] vs. 1.8 [1.4–2.1]), indicating attenuation of the genotype difference in insulin sensitivity. Additional findings: No overall dietary effect was observed on OGTT-derived outcomes or HbA1c (β = 0.04, 95% CI −0.01 to 0.09; *p* = 0.136). No genotype × diet interactions were observed for continuous glucose monitoring outcomes.
Mahmoudinezhad et al., 2023 ([81])	Cross-Sectional	347 (58.2/41.8)	40.8 ± 9.2	Iran	*FADS2* rs174583	0.24	NA	HEI and DQI-I	Serum glucose; insulin; HOMA-IR; QUICKI	Formal interaction test: Significant HEI × FADS2 rs174583 interactions were observed for insulin (*p* < 0.001), HOMA-IR (*p* < 0.001), and QUICKI (*p* = 0.001). Main finding: TT homozygotes exhibited the highest insulin and HOMA-IR levels even among participants with high adherence to the HEI, indicating a less favorable insulin-related metabolic profile than C-allele carriers despite better diet quality.
Ramos-Lopez et al., 2019 ([82])	Cross-Sectional	179 (72/28)	45.3 ± 0.7	Caucasian	95-SNP GRS	NA	Y	Dietary intake (% energy from carbohydrates [simple/complex], protein [animal/plant], fat [SFA/MUFA/PUFA]; plus dietary cholesterol, fiber, water)	Glucose; insulin; HOMA-IR	Formal interaction test: Significant interaction between weighted genetic risk score (wGRS) and dietary cholesterol for HOMA-IR (Pinteraction = 0.002). Main finding: Higher dietary cholesterol was associated with higher HOMA-IR only among participants with a high wGRS (*p* < 0.001), whereas no association was observed in those with a low wGRS (*p* = 0.185).
Noumi et al., 2018 ([83])	Cross-Sectional	1981 (36/64)	30–79	Japanese	*RETN* rs1862513	NA	NA	Nutrient intake by FFQ (quartiles), focus on *n*-3 PUFA (also fish/meat)	Serum resistin	Formal interaction test: Significant genotype × n-3 PUFA interaction (Pinteraction = 0.004). Main finding: Serum resistin decreased across increasing dietary n-3 PUFA quartiles (Q1–Q4: 12.5, 12.5, 12.2, and 11.5 ng/mL; Ptrend = 0.007), with the strongest inverse association observed in GG homozygotes (Q1–Q4: 18.9, 19.5, 18.4, and 14.5 ng/mL; Ptrend = 0.001). Additional findings: The inverse association remained significant in CG (Ptrend = 0.015) and CC carriers (Ptrend = 0.020), although the magnitude of the association was smaller.
de Luis et al., 2017b [84]	Non-randomized intervention	80 (26.3/73.7)	47.2 ± 9.3	Caucasian	*BDNF* rs10767664	0.22	Y	3-month standard hypocaloric diet	fasting insulin; HOMA-IR	Formal interaction test: Not reported. Main finding: Following the 3-month hypocaloric diet, non-T allele carriers showed greater reductions in fasting insulin (−2.1 ± 1.9 vs. −0.3 ± 1.0 mIU/L; *p* = 0.01) and HOMA-IR (−0.9 ± 0.4 vs. −0.1 ± 0.8; *p* = 0.01) than T-allele carriers.
de Luis et al., 2017c [85]	Non-randomized intervention	82 (24.4/75.6)	47.1 ± 10.3	Caucasian	*ADIPOQ* rs1501299	0.27	Y	3-month standard hypocaloric diet	Fasting glucose; insulin; HOMA-IR	Formal interaction test: Not reported. Main finding: Following the 3-month hypocaloric diet, GG homozygotes exhibited greater reductions in fasting glucose (−4.8 ± 2.5 vs. −0.5 ± 0.1 mg/dL; *p* = 0.02), fasting insulin (−3.6 ± 1.5 vs. +0.6 ± 1.1 mIU/L; *p* = 0.02), and HOMA-IR (−1.2 ± 0.9 vs. −0.1 ± 1.1; *p* = 0.03) than GT/TT carriers.
Franck et al., 2019 ([86])	Non-randomized intervention	210 (NA)	High risk: 28.2 ± 8.4 Low risk: 32.6 ± 8.8	Canada	*ADGRL2* rs72723587; *LOC101929563* rs77850702; *LOC101929563* rs72703546; *PTPRO* rs17174795; *TPM1* rs12437986; *RPH3AL* rs35621498; *RPH3AL* rs55842940; *TNRC6B* rs6001872	rs72723587 A 0.19; rs77850702 G 0.15; rs72703546 C 0.13; rs17174795 C 0.12; rs12437986 A 0.24; rs35621498 A 0.58; rs55842940 A 0.44; rs6001872 G 0.39	Y	6-week *n*-3 PUFA supplementation: 5 g/day fish oil (EPA 1.9–2.2 g + DHA 1.1 g/day)	HOMA-IR (pre vs. post); high-risk vs. low-risk classification by HOMA-IR change	Formal interaction test: Not reported. Main finding: Following 6-week n-3 PUFA supplementation, the genetic risk score was associated with an increased likelihood of HOMA-IR deterioration (OR = 3.16, 95% CI 1.85–7.14) in the testing cohort. Additional findings: Overall, 23.2% (32/138) of participants exhibited increased HOMA-IR after supplementation. The genetic model explained approximately 40% of the variation in HOMA-IR response and achieved a predictive accuracy of 0.85.
Huang et al., 2011 ([87])	Non-randomized intervention	56 (48.2/51.8)	22.9 ± 1.8	China	*LPL* rs328; rs320	rs328: 0.12; rs320: 0.25	Y	Control diet 7 days (30.1% fat, 54.1% carbohydrate) then HC/LF diet 6 d (13.8% fat, 70.1% carbohydrate); similar fatty-acid composition; no energy restriction	HOMA IR, Glucose, Insulin	Formal interaction test: No formal genotype × diet interaction test reported. Main finding: Following the high-carbohydrate/low-fat diet, female S447S homozygotes showed significant increases in fasting insulin (4.22 ± 2.78 → 6.02 ± 3.17 μU/mL; *p* < 0.01) and HOMA-IR (0.84 ± 0.59 → 1.17 ± 0.65; *p* < 0.01), whereas female 447X carriers showed no significant changes. Additional findings: Among males, 447X carriers showed lower fasting glucose after the intervention (87.21 ± 6.54 → 77.24 ± 7.38 mg/dL; *p* < 0.05). Similar sex-specific findings were observed for the HindIII polymorphism.
Goyenechea et al., 2009 ([88])	Non-randomized intervention	180 (51.7/48.3)	35.0 ± 5.0	Spain	*ADIPOQ* rs17300539	0.12	Y	8-week calorie-restricted balanced diet (55% carbohydrate, 15% protein, 30% fat; ~30% energy restriction), then weight-maintenance period with dietary guidance (no calorie restriction)	Glucose, Insulin, HOMA-IR	Formal interaction test: Not reported. Main finding: Following the 8-week calorie-restricted intervention, improvements in fasting glucose, insulin, and HOMA-IR did not differ according to ADIPOQ rs17300539 genotype. Additional findings: During the 60-week weight-maintenance period, GG homozygotes exhibited a greater rebound in HOMA-IR than A-allele carriers (genotype effect *p* = 0.037). HOMA-IR increased significantly in GG homozygotes (*p* < 0.001) but not in A-allele carriers (*p* = 0.317). GG homozygotes also regained weight (+1.4 ± 1.0 kg; *p* = 0.021), whereas A-allele carriers showed no significant weight regain (−0.3 ± 0.7 kg; *p* = 0.271).
AlSaleh et al., 2013 ([89])	RCT	312 (38.1/61.9)	55.0 ± 7.0	Mixed	*ADIPOQ* rs17300539, rs266729, rs2241766, rs1501299	rs17300539 (A) = 0.08; rs266729 (G) = 0.27; rs2241766 (G) = 0.14; rs1501299 (T) = 0.27	Y	4-week run-in: olive-oil placebo capsules + restrict oily fish; then 12 months: EPA + DHA supplements (0.45, 0.9, or 1.8 g/d; 1.5:1) vs. placebo; oily fish limited to 1 portion/month	Glucose, Insulin, HOMA-IR	Formal interaction test: No significant genotype × treatment interactions were observed for fasting glucose, insulin, or HOMA-IR. Main finding: n-3 PUFA supplementation did not modify fasting glucose, insulin, or HOMA-IR according to ADIPOQ genotype. Additional findings: A nominal rs2241766 × treatment × age interaction was observed for adiponectin (*p* = 0.029), with TT homozygotes aged >58 years showing an approximately 22% increase in adiponectin following 1.8 g/day EPA + DHA, whereas no comparable response was observed in G-allele carriers.
Moradi et al., 2018 ([90])	Cross-Sectional	288 (0/100)	38.5 ± 12.1	Iran	*PPARGC1A* rs11290186, rs2970847	0.42	Y	Dietary fat intake from 3-day 24 h recalls; fat %E categorized vs. RDA (30% energy) and SFA vs. RDA (7% energy)	Insulin	Formal interaction test: No significant genotype × dietary fat interactions were observed. Main finding: Fasting insulin differed according to genotype for rs2970847 (*p* = 0.004) and rs11290186 (*p* = 0.03), independent of dietary fat intake.

AIRg, acute insulin response to glucose; AUC, area under the curve; BIGTT-AIR, BIGTT-derived acute insulin response; BIGTT-Si, BIGTT-derived insulin sensitivity index; BMI, body mass index; β, beta coefficient CGM; continuous glucose monitoring; DHA, docosahexaenoic acid; DQI-I, Diet Quality Index-International; EPA, eicosapentaenoic acid; FFA, free fatty acids; FFQ, food frequency questionnaire; FPG, fasting plasma glucose; GI, glycemic index; HC/LF, high-carbohydrate/low-fat; HEI, Healthy Eating Index; HF, high-fat; HOMA-S, Homeostatic Model Assessment of insulin sensitivity; HP, high-protein; iAUC, incremental area under the curve; IFG, impaired fasting glucose; ISI_0–120_, insulin sensitivity index from 0 to 120 min; ivGTT, intravenous glucose tolerance test; LCD, low-calorie diet; LF, low-fat; NEFA, non-esterified fatty acids; P:S, polyunsaturated-to-saturated fat ratio; Q, quartile; QUICKI, quantitative insulin sensitivity check index; Padd, *p* value under the additive genetic model; Pdom, *p* value under the dominant genetic model; Pint, *p* value for interaction; Ptrend, *p* value for trend; RDA, recommended dietary allowance; SE, standard error; SSPG, steady-state plasma glucose; SSPI, steady-state plasma insulin; wGRS, weighted genetic risk score; WHO, World Health Organization.

**Table 4:** Gene–diet interactions and glycemic traits/β-cell function.

Study	Study Design	No. of Participants—All (M/F%)	Age (Years, Range/Mean)	Region, Ethnicity/Nationality	Gene(s)—SNP(s)	EAF	HWE Met	Intervention/Exposure	Outcome (Measure)	Result
A. Macronutrient intake										
Bauer et al., 2021 ([91])	Cross-Sectional	810 (47.5/52.5)	42.1 ± 14.5	Caucasian	*TCF7L2* rs7901695	0.26	Y	Macronutrient intake categories (quantiles): protein ≤ 18% vs. >18% energy; carbohydrate ≤48% vs. >48% energy; fat ≤ 30% vs. >30% energy	OGTT glucose/insulin (0, 30, 60, 120 min); HbA1c; HOMA-IR; corrected insulin response (CIR30)	Formal interaction test: Significant protein × TCF7L2 rs7901695 interaction for HbA1c (β = 0.37, 95% CI 0.01–0.74; *p* = 0.05). Main finding: Among TT homozygotes, higher protein intake (>18% energy) was associated with higher HbA1c (β = 0.37, 95% CI 0.01–0.74; *p* = 0.05), higher HOMA-IR (*p* = 0.014), higher fasting insulin (*p* = 0.03), and higher glucose and insulin responses during the OGTT (*p* ≤ 0.012 and *p* = 5.7 × 10^−5^, respectively). Additional findings: Among TT homozygotes, lower carbohydrate intake (≤48% energy) was associated with higher HbA1c (*p* = 0.041) and lower CIR30 (*p* = 0.032).
Mattei et al., 2012 ([92])	RCT	591 (43.2/56.8)	30–70	Caucasian	*TCF7L2* rs7903146, rs12255372	s7903146 (T) = 0.29; rs12255372 (T) = 0.28	Y	Energy-reduced diets (−750 kcal/day) randomized to 4 macronutrient targets; primary comparison for gene–diet interaction: low-fat (20% energy) vs. high-fat (40% energy) (secondary: low vs. high protein; extreme carbohydrates), 2 years	Fasting glucose and insulin	Formal interaction test: Not reported Main finding: Among rs12255372 TT homozygotes, weight loss predicted greater reductions in fasting glucose and insulin only in the low-fat diet: Δweight predicted Δglucose (β = 0.58, 95% CI 0.20–0.97; *p* = 0.006) and Δinsulin (β = 0.47, 95% CI 0.14–0.81; *p* = 0.009), whereas these associations were not significant in the high-fat diet (glucose *p* = 0.362; insulin *p* = 0.265). Additional findings: No genotype differences were observed in baseline glycaemic measures or in overall changes at 6 months or 2 years.
B. Food-specific dietary exposures										
Ouhaibi-Djellouli et al., 2014 ([93])	Cross-Sectional	787(48/52)	42.8 ± 9.6	Algeria	*TCF7L2* rs7903146	0.43	Y	FFQ-based classification of participants as low vs. moderate/high consumers using the median intake for each food.	Fasting glucose; HOMA-IR; HOMA-B	Formal interaction test: Significant dessert intake × TCF7L2 rs7903146 interaction for fasting glucose (Pinteraction = 0.02). Main finding: Among non-diabetic participants consuming ≥1 dessert/day, the T allele was associated with higher fasting glucose concentrations (*p* = 0.03), whereas no genotype effect was observed among those with lower dessert intake. Additional findings: Dessert intake also modified the association between TCF7L2 rs7903146 and type 2 diabetes risk. Among participants consuming ≥1 dessert/day, T-allele carriers had 2.61-fold higher odds of T2D (OR = 2.61, 95% CI 1.51–4.52; *p* = 0.0006), whereas no increased risk was observed among low dessert consumers. A similar interaction was observed for milk intake and T2D risk (Pinteraction = 0.01).
Wang et al., 2021 ([94])	Case–Control	4202 (51.6/48.4)	56.9 ± 11.0	China	*INAFM2* rs67839313	0.3	Y	Egg intake grouped by median: <4 eggs/week vs. ≥4 eggs/week	Fasting glucose	Formal interaction test: Significant egg intake × INAFM2 rs67839313 interaction for fasting glucose (Pinteraction = 0.003). Main finding: The T allele was associated with a 0.188 mmol/L increase in fasting glucose among participants consuming <4 eggs/week, but a 0.152 mmol/L decrease among those consuming ≥4 eggs/week.
Ortega-Azorín et al., 2012 ([95])	Case–Control	3192 (NA)	55–80	Caucasian	*FTO* rs9939609; *MC4R* rs17782313	*FTO* rs9939609: 0.18 *MC4R* rs17782313: 0.05	Y	Mediterranean diet adherence score; folate intake (low <406 µg/day vs. high ≥406 µg/day)	Fasting glucose	Formal interaction test: Significant folate × FTO rs9939609 interaction for fasting glucose (Pinteraction = 0.023). Main finding: Among non-diabetic participants with low folate intake (<406 μg/day), carriers of the FTO rs9939609variant allele had higher fasting glucose concentrations than wild-type individuals, whereas this genotype difference was not observed among those with higher folate intake. Additional findings: The interaction remained significant after adjustment for waist circumference (Pinteraction = 0.018) and dietary fiber (Pinteraction = 0.028). A significant aggregate genetic score × folate interaction was also observed (Pinteraction = 0.026; 0.021 after adjustment for waist circumference; 0.047 after adjustment for dietary fibre).
C. Other diet–gene associations										
Enhörning et al., 2009 ([96])	Cross-Sectional	6055 (42.2/57.8)	57.5 ± 5.9	Caucasian	*AVPR1A* rs1042615, rs10784339, rs7308855, rs10747983	rs1042615 T = 0.45; rs10747983 A = 0.16; rs10784339 C = 0.16; rs7308855 T = 0.09	Y	Dietary fat intake quartiles (Q1–Q4; energy-adjusted residual method)	Fasting glucose	Formal interaction test: Significant dietary fat × AVPR1A rs1042615 interaction for fasting glucose in men (Pinteraction = 0.044). Main finding: In men within the highest dietary fat intake quartile, T-allele carriers (CT/TT) had higher fasting glucose than CC homozygotes (5.27 vs. 5.13 mmol/L; adjusted *p* = 0.061). Additional findings: No genotype differences were observed in lower fat-intake quartiles or among women. No associations were identified for the other AVPR1A polymorphisms.
Tsuzaki et al., 2009 ([97])	Non-randomized intervention	32 (0/100)	50.0 ± 8.0	Japanese	*ADIPOQ* rs266729	0.29	Y	2 months low-calorie diet (5023 kJ/day) with dinner meal replacement; habitual lifestyle maintained	Fasting glucose	Formal interaction test: Not reported. Main finding: Following the 2-month low-calorie diet, G-allele carriers (CG/GG) exhibited greater reductions in fasting glucose than CC homozygotes (−7 ± 8 vs. −2 ± 4 mg/dL; *p* = 0.044).

CIR30, corrected insulin response at 30 min; FBG, fasting blood glucose.

#### 3.4.2. Genetic Variants Interacting with Macronutrient Intake

Five cohort studies examined interactions between genetic variants and macronutrient intake in relation to incident T2D (Table 2B). In a Korean cohort, energy intake modified the association between a 6-SNP GRS and T2D, with stronger associations observed among individuals with higher energy intake [41]. However, no significant interactions were observed for carbohydrate, protein, fat, or calcium intake.

In another Korean cohort, carbohydrate intake did not significantly interact with a 10-SNP GRS in relation to T2D risk, although stratified analyses suggested stronger genetic associations among individuals consuming lower-carbohydrate diets [42].

Evidence of gene–macronutrient interactions was reported in a large Swedish cohort, where *GIPR* rs10423928 interacted with both carbohydrate and fat intake in relation to incident T2D [43]. Higher carbohydrate intake was associated with lower T2D risk among TT individuals, whereas higher fat intake was associated with reduced risk among AA individuals.

In the European Prospective Investigation into Cancer and Nutrition (EPIC)-Potsdam cohort, *CAV2* rs2270188 interacted with total fat and saturated fat intake, with higher fat and SFA intake associated with increased T2D risk among TT homozygotes [44].

Finally, a Swedish cohort study reported sex-specific interactions between *IRS1* rs2943641 and macronutrient intake. The protective T allele was associated with lower T2D risk among women consuming low or moderate carbohydrate diets and among men consuming low-fat diets [45].

#### 3.4.3. Gene–Food Interactions

Four studies examined interactions between genetic variants and specific foods or dietary components in relation to incident T2D (Table 2C). Two cohort studies reported interactions between *TCF7L2* rs7903146 and fiber or whole-grain intake. In a Swedish cohort, higher fiber intake was associated with lower T2D risk only among individuals with the CC genotype, whereas no protective association was observed among T-allele carriers [46]. Similarly, in the EPIC-Potsdam study, whole-grain intake was inversely associated with T2D risk in CC individuals but not in carriers of the T allele [47].

A randomized controlled trial from the Prevención con Dieta Mediterránea (PREDIMED) study evaluated the *CLOCK* rs4580704 variant and adherence to a Mediterranean diet. G-allele carriers had a lower incidence of T2D compared with CC individuals; however, the formal interaction between genotype and diet assignment was not statistically significant [48].

Finally, a cohort study reported a nominal interaction between *FFAR4* rs116454156 and dietary fat intake, in which carriers of the R270H variant had increased T2D risk under low-fat intake, while no differences were observed in higher fat intake categories [49].

### 3.5. Gene–Diet Interactions for Insulin Resistance/Insulin Sensitivity/Insulin-Related Traits

#### 3.5.1. Macronutrient Composition in Energy-Restricted Diets

Eighteen studies evaluated whether genetic variants modify metabolic responses to energy-restricted diets differing in macronutrient composition (Table 3A). Interactions with dietary fat or carbohydrate intake were frequently reported. For example, *PPM1K* rs1440581 modified responses to low-fat versus high-fat diets, with T-allele carriers showing smaller improvements in insulin resistance on low-fat/high-carbohydrate diets but greater improvements on high-fat/low-carbohydrate diets [52,60]. Similarly, *TCF7L2* rs7903146 influenced changes in insulin resistance during weight-loss interventions, with TT individuals experiencing smaller reductions in HOMA-IR on high-fat diets compared with low-fat diets [62].

Interactions with dietary carbohydrate were also observed. *PCSK7* rs236918 modified responses to diets differing in carbohydrate content, with greater reductions in fasting insulin among G-allele carriers assigned to high-carbohydrate diets [51]. The *IRS1* rs2943641 variant similarly interacted with macronutrient composition, with CC individuals showing greater improvements in insulin and HOMA-IR on higher-carbohydrate diets [66]. In addition, *GIPR* rs2287019 modified responses to dietary fat intake, with greater reductions in fasting glucose, insulin, and HOMA-IR among T-allele carriers assigned to low-fat diets at 6 months [61].

Other studies reported genotype-dependent responses to hypocaloric diets involving variants in *BDNF*, *MTNR1B*, *FTO*, *HNF1A*, and genes related to vitamin D metabolism (*DHCR7*), influencing changes in insulin resistance or related metabolic markers [50,58,59,64,67]. Additional studies also reported differential responses according to variants in *ACE*, *UCP3*, *ADIPOQ*, and *FTO*, as well as a 31-SNP GRS, in relation to fasting glucose, insulin, HOMA-IR, HOMA-B, or HbA1c during energy-restricted interventions [54,55,56,57,63]. Genetic risk scores were also investigated; both an 8-SNP GRS and a 14-SNP GRS showed interactions with dietary fat intake for changes in insulin resistance markers at 6 months, although these effects were attenuated over longer follow-up [53,65].

Overall, gene–diet interactions on insulin resistance responses to weight-loss diets were reported across multiple loci, although these effects were often short-term and varied by dietary macronutrient composition.

#### 3.5.2. Fat Quality and Mediterranean-Type Diets

Eleven studies examined whether genetic variants modify metabolic responses to diets differing in fat quality, including saturated (SFA), monounsaturated (MUFA), and polyunsaturated fatty acids (PUFA), as well as Mediterranean-type dietary patterns (Table 3B). In crossover trials, switching from SFA-rich diets to MUFA- or carbohydrate-rich diets improved insulin sensitivity in some genotype groups. For example, *APOE* rs405509 influenced responses to fat quality, with reductions in steady-state plasma glucose observed in GG and GT individuals but not in TT carriers following MUFA- or carbohydrate-rich diets [68]. Similarly, *FABP2* rs1799883 interacted with dietary fat composition, with Thr54 carriers showing higher insulin resistance after SFA-rich diets compared with MUFA or carbohydrate-rich diets [69].

Other studies reported genotype-dependent responses involving genes related to lipid metabolism and adiposity. Variants in *ADIPOQ* were associated with differential changes in insulin resistance during hypocaloric diets enriched in MUFA or PUFA [70], while *PPARγ* rs1801282 interacted with the dietary polyunsaturated-to-saturated fat ratio in relation to fasting insulin levels [73]. Interactions with Mediterranean-type diets were also observed for variants in *LEPR*, *APOB*, *SCARB1*, *LIPC* and *IRS1*, influencing measures of insulin sensitivity or insulin resistance in several crossover feeding trials [74,75,76,77,78]. Additional sex-specific diet–genotype interactions were also reported for *ADIPOQ* variants in a crossover feeding study [72]. In addition, *FABP2* rs1799883 was associated with differences in postprandial insulin responses following consumption of oils differing in fatty acid composition [71].

Overall, these findings suggest that genetic variation may influence metabolic responses to dietary fat quality and Mediterranean-type dietary patterns.

#### 3.5.3. Other Dietary Exposures

Twelve studies examined gene–diet interactions involving dietary factors beyond macronutrient composition or fat quality (Table 3C). In a cross-sectional study, *PLIN1* rs894160 interacted with carbohydrate intake in relation to HOMA-IR, although the genotype main effect was not significant [79]. A crossover trial in Greenland Inuit participants reported an interaction between *TBC1D4* rs61736969 and dietary pattern for insulin sensitivity indices, with differences between variant carriers and non-carriers persisting after a traditional Inuit diet but attenuated following a Western diet [80].

Diet quality indices were also investigated. In an Iranian cohort, *FADS2* rs174583 interacted with the HEI and DQI-I in relation to insulin-related traits, with TT carriers showing higher insulin and HOMA-IR despite higher diet quality [81]. In addition, a 95-SNP genetic risk score interacted with dietary cholesterol intake for HOMA-IR, with higher cholesterol intake associated with greater insulin resistance among individuals with higher genetic risk [82].

Other studies examined interactions involving specific nutrients or dietary interventions. *RETN* rs1862513 modified the association between n−3 PUFA intake and circulating resistin concentrations [83]. Improvements in insulin resistance following hypocaloric diets differed according to variants in *BDNF*, *ADIPOQ* and *LPL* in short-term interventions [50,84,87], and HOMA-IR rebound during the weight-maintenance phase also differed according to *ADIPOQ* rs17300539 [88]. A gene-based analysis of n−3 PUFA supplementation identified several loci associated with changes in HOMA-IR and constructed a genetic risk score predictive of insulin resistance response [86]. In contrast, no significant treatment–genotype interactions were observed for insulin resistance outcomes following long-term n−3 PUFA supplementation in a randomized trial examining *ADIPOQ* variants [89], and dietary fat intake did not significantly modify associations between *PPARGC1A* variants and insulin levels [90].

### 3.6. Gene–Diet Interactions and Glycemic Traits/β-Cell Function

#### 3.6.1. Macronutrient Intake

Two studies examined interactions between genetic variants and macronutrient intake in relation to glycemic traits and β-cell function (Table 4A). In a cross-sectional study, *TCF7L2* rs7901695 interacted with dietary protein intake in relation to glycemic markers. Individuals with the TT genotype consuming higher protein diets (>18% energy) had higher HbA1c compared with CT and CC carriers, and among TT carriers, higher protein intake was associated with higher HOMA-IR and greater glucose and insulin responses during oral glucose tolerance testing [91]. Lower carbohydrate intake was also associated with lower corrected insulin response among TT carriers.

In a randomized controlled trial of energy-restricted diets differing in macronutrient composition, *TCF7L2* rs12255372 modified glycemic responses to weight loss. Decreases in fasting glucose and insulin associated with reductions in body weight or BMI were observed in G-allele carriers across both low-fat and high-fat diets, whereas in individuals with the TT genotype these improvements were observed primarily in the low-fat diet group [92].

Together, these studies reported interactions between *TCF7L2* variants and macronutrient intake in relation to glycemic outcomes.

#### 3.6.2. Food-Specific Dietary Exposures

Three studies examined interactions between genetic variants and specific foods or dietary components in relation to glycemic traits (Table 4B). In a cross-sectional study, the *TCF7L2* rs7903146 variant interacted with dessert consumption in relation to fasting glucose, with the T allele associated with higher fasting glucose only among individuals consuming at least one dessert per day [93].

In a case–control study, *INAFM2* rs67839313 interacted with egg intake in relation to fasting blood glucose. The T allele was associated with higher fasting glucose among individuals consuming fewer than four eggs per week but with lower fasting glucose among those consuming four or more eggs per week [94].

Another case–control study reported an interaction between *FTO* rs9939609 and folate intake, where variant-allele carriers with low folate intake had higher fasting glucose levels compared with wild-type individuals, whereas this difference was not observed among those with higher folate intake [95]. A significant interaction was also observed between folate intake and a combined genetic score, including *FTO* and *MC4R* variants.

#### 3.6.3. Other Gene–Diet Associations

Two studies reported additional gene–diet interactions related to glycemic traits (Table 4C). In a cross-sectional study, *AVPR1A* rs1042615 interacted with dietary fat intake in relation to fasting glucose among men, with T-allele carriers in the highest fat-intake [96].

In a dietary intervention study, *ADIPOQ* rs266729 modified the glycemic response to a low-calorie diet. Individuals carrying the G allele experienced greater reductions in fasting glucose following a two-month calorie-restricted diet compared with CC homozygotes [97].

### 3.7. Risk-of-Bias Assessment

**Figure 2:**
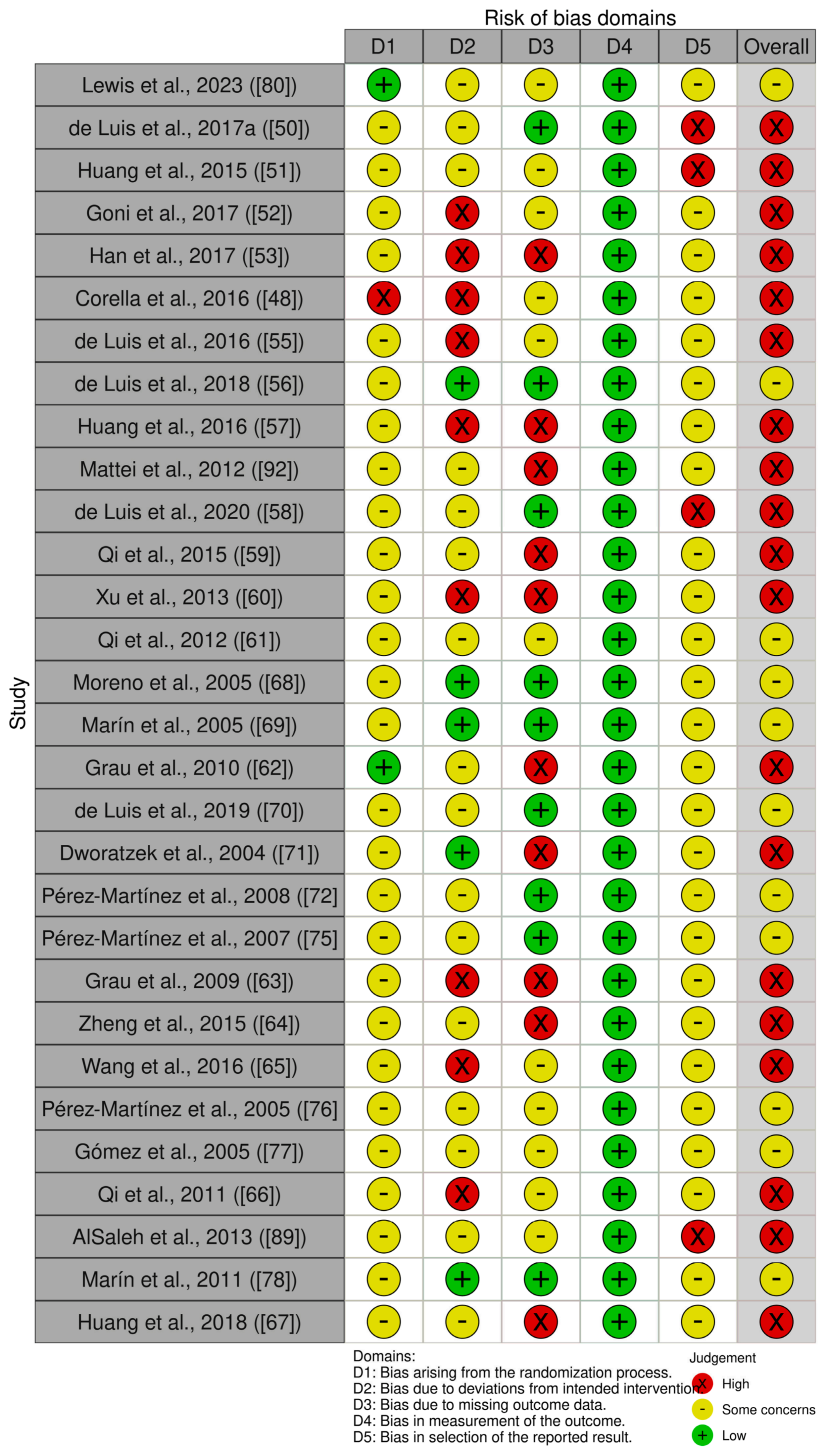
Risk-of-bias assessment using the RoB 2 tool for crossover and parallel-group RCTs. The majority of RCTs (63.33%, *n* = 19) were classified as having high overall risk of bias, while 11 studies (36.67%) were judged to raise some concerns overall. High overall risk of bias was attributable to bias arising from the randomization process (*n* = 1), deviations from intended interventions (*n* = 9), missing outcome data (*n* = 10), and the selection of the reported result (*n* = 4). At the domain level, judgments of some concerns were more frequent for the randomization process (*n* = 27), followed by selection of the reported result (*n* = 26), deviations from intended interventions (*n* = 16), and missing outcome data (*n* = 11). Visualization generated using the Risk-of-bias VISualization (robvis) web application (University of Bristol, Bristol, UK; accessed February 2026) [48,50,51,52,53,55,56,57,58,59,60,61,62,63,64,65,66,67,68,69,70,71,72,75,76,77,78,80,89,92,98].

**Figure 3:**
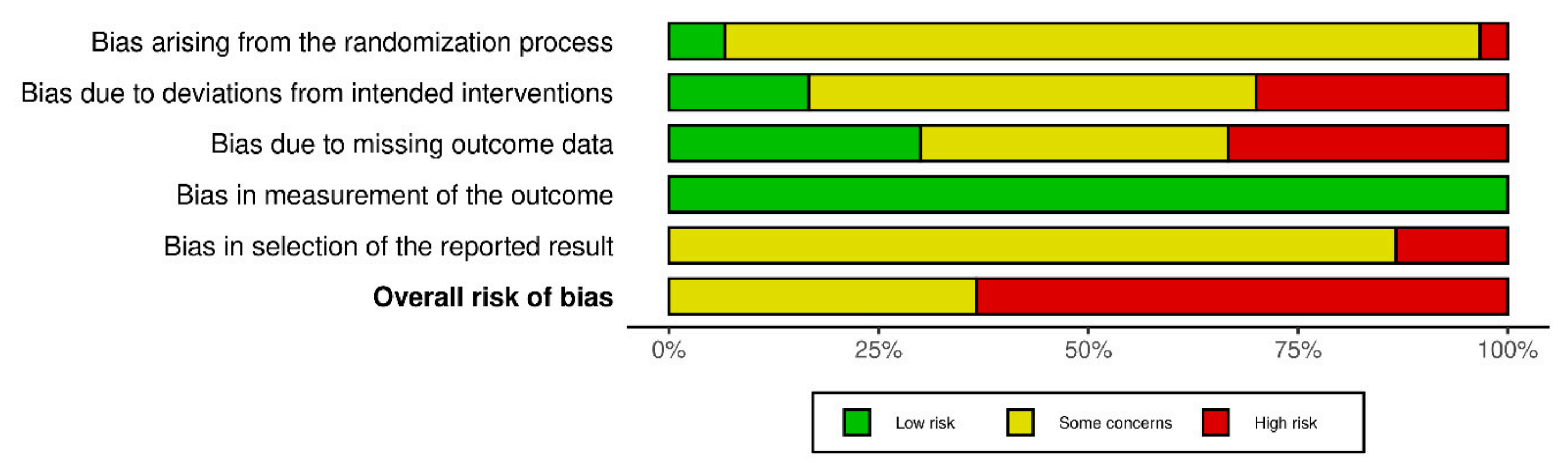
Summary of risk-of-bias assessment for randomized controlled trials (crossover RCTs, *n* = 8; parallel-group RCTs, *n* = 22; total *n* = 30). Bias arising from the randomization process (low risk [*n* = 2, 6.67%], some concerns [*n* = 27, 90.00%], high risk [*n* = 1, 3.33%]); bias due to deviations from intended interventions (low risk [n = 5, 16.67%], some concerns [*n* = 16, 53.33%], high risk [*n* = 9, 30.00%]); bias due to missing outcome data (low risk [*n* = 9, 30.00%], some concerns [*n* = 11, 36.67%], high risk [*n* = 10, 33.33%]); bias in measurement of the outcome (low risk [*n* = 30, 100.00%]); bias in selection of the reported result (some concerns [*n* = 26, 86.67%], high risk [*n* = 4, 13.33%]); overall risk of bias (some concerns [*n* = 11, 36.67%]; high risk [*n* = 19, 63.33%]). Visualization generated using the robvis web application (University of Bristol, Bristol, UK; accessed February 2026) [98].

**Figure 4:**
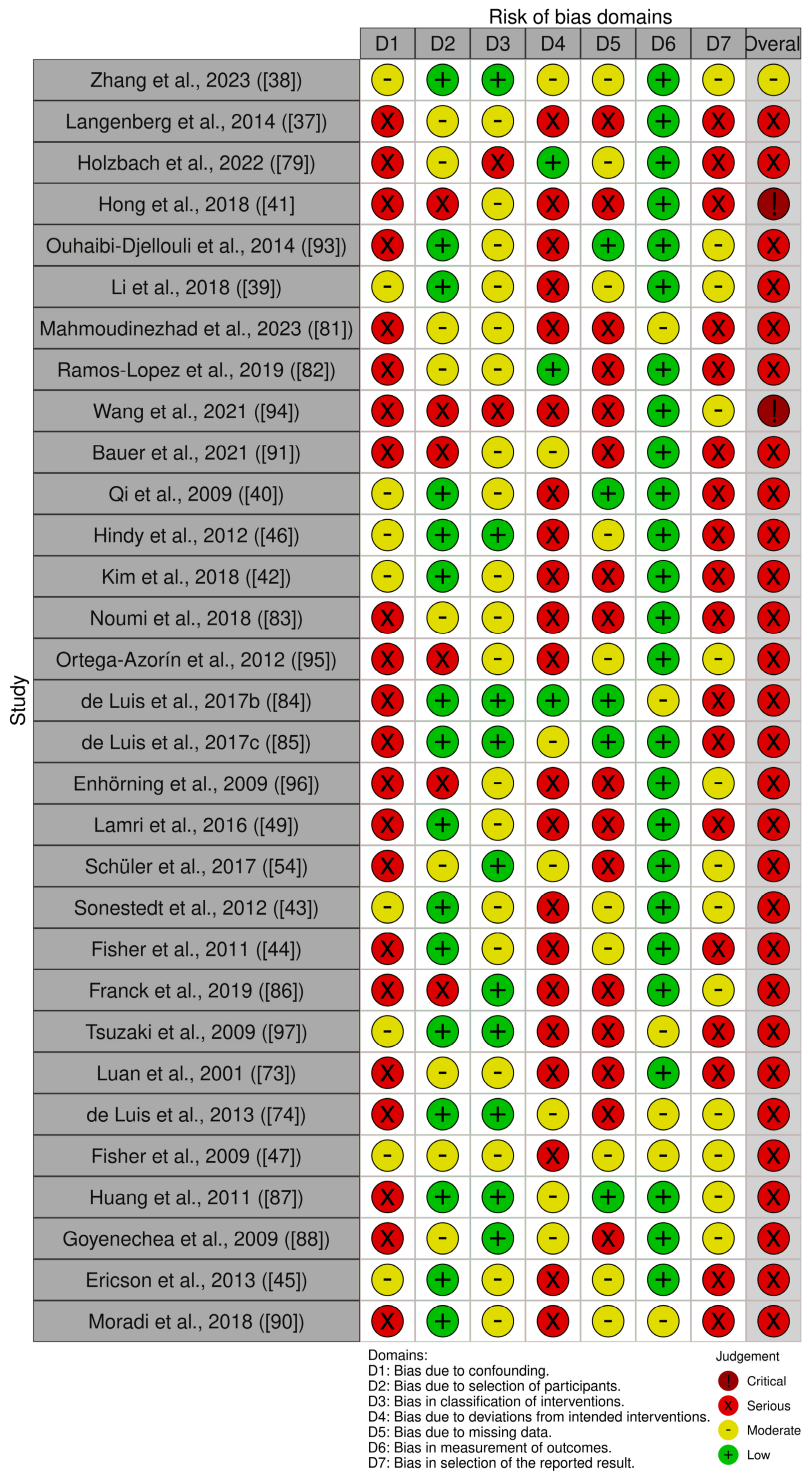
Risk-of-bias assessment using the ROBINS-I for non-randomized studies of interventions. Overall, 1 study (3.23%) demonstrated moderate risk of bias, 28 studies (90.32%) demonstrated a serious risk of bias, while 2 studies (6.45%) demonstrated a critical risk of bias. Bias due to confounding was the domain most frequently judged as serious (*n* = 22, 70.97%), followed by bias due to deviations from intended interventions (*n* = 21, 67.74%), bias in selection of the reported result (*n* = 18, 58.06%), and bias due to missing data (*n* = 16, 51.61%). In contrast, bias in measurement of outcomes was most frequently judged as low risk (*n* = 25, 80.65%). Bias in classification of interventions was most often rated as moderate (*n* = 19, 61.29%), whereas bias due to selection of participants was predominantly judged as low risk (*n* = 16, 51.61%). Visualization generated using the robvis web application (University of Bristol, Bristol, UK; accessed February 2026) [37,38,39,40,41,42,43,44,45,46,47,49,54,73,74,79,81,82,83,84,85,86,87,88,90,91,93,94,95,96,97,98].

**Figure 5:**
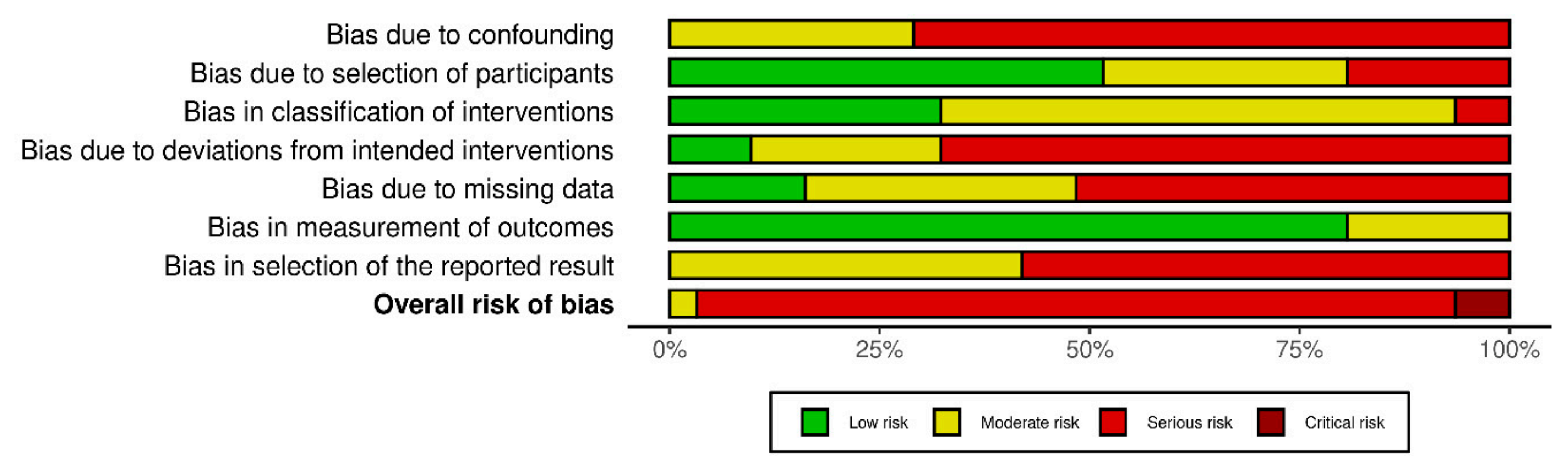
Summary of risk-of-bias assessment for non-randomized studies of interventions (*n* = 31). Bias due to confounding (moderate risk [*n* = 9, 29.03%], serious risk [*n* = 22, 70.97%]); bias due to selection of participants (low risk [*n* = 16, 51.61%], moderate risk [*n* = 9, 29.03%], serious risk [*n* = 6, 19.36%]); bias in classification of interventions (low risk [*n* = 10, 32.26%], moderate risk [*n* = 19, 61.29%], serious risk [*n* = 2, 6.45%]); bias due to deviations from intended interventions (low risk [*n* = 3, 9.68%], moderate risk [*n* = 7, 22.58%], serious risk [*n* = 21, 67.74%]); bias due to missing data (low risk [*n* = 5, 16.13%], moderate risk [*n* = 10, 32.26%], serious risk [*n* = 16, 51.61%]); bias in measurement of outcomes (low risk [*n* = 25, 80.65%], moderate risk [*n* = 6, 19.35%]); bias in selection of the reported result (moderate risk [*n* = 13, 41.94%], serious risk [*n* = 18, 58.06%]); overall risk of bias (moderate risk [*n* = 1, 3.23%], serious risk [*n* = 28, 90.32%]; critical risk [*n* = 2, 6.45%]). Visualization generated using the robvis web application (University of Bristol, Bristol, UK; accessed February 2026) [98].

The overall and domain risk-of-bias assessment results are displayed in Figure 2, Figure 3, Figure 4 and Figure 5.

## 4. Discussion

This systematic review included 61 reports examining gene–diet interactions in relation to incident T2D, insulin-related traits, and glycemic outcomes among adults without diagnosed T2D at baseline. Reported interactions spanned dietary patterns, macronutrient composition, dietary fat quality, and selected food-based exposures, with *TCF7L2*, *IRS1*, *GIPR*, *ADIPOQ*, *FTO*, and genetic risk scores among the most frequently investigated factors. However, the evidence base was heterogeneous across study design, dietary assessment, genetic modeling, and outcome definitions, and several reported interactions were identified in single studies without independent replication. These considerations limit confidence in the consistency and clinical applicability of the current evidence. Across the reviewed literature, the most repeatedly studied and comparatively more coherent signals were observed for *TCF7L2* in relation to fiber/whole-grain or macronutrient exposures, and for several loci examined in short-term weight-loss interventions differing in macronutrient composition; however, even these findings require stronger independent replication.

### 4.1. Gene–Diet Interactions and Incident T2D Risk

Evidence for gene–diet interactions in relation to incident T2D was mixed and appeared to differ depending on whether studies examined broad dietary patterns or specific dietary exposures. The clearest evidence for an interaction between overall dietary pattern and genetic susceptibility was reported by Qi et al. (2009) [40], who showed that adherence to a Western dietary pattern amplified T2D risk among individuals with higher genetic susceptibility. In contrast, other large cohort studies did not observe significant interactions between genetic risk scores and adherence to healthier dietary patterns, including the Mediterranean diet and EAT–Lancet diet index (Langenberg et al., 2014; Zhang et al., 2023) [37,38]. Similarly, Li et al. (2018) [39] found no evidence that macronutrient composition modified the association between polygenic risk and incident T2D. Taken together, these findings suggest that interactions between overall diet quality and polygenic susceptibility are likely modest and not consistently detectable across cohorts.

By contrast, somewhat more specific signals were reported for individual loci in relation to macronutrient intake. For example, Sonestedt et al. (2012) [43] observed significant interactions between *GIPR* rs10423928 and carbohydrate and fat intake, while other studies reported interactions involving *CAV2* and *IRS1* in relation to fat or carbohydrate exposure. However, these findings were locus-specific, not widely replicated, and in some cases differed by sex or dietary metric. Overall, the available evidence suggests that gene–diet interactions influencing incident T2D risk may be more detectable for selected candidate loci and specific dietary exposures than for broad measures of overall diet quality.

### 4.2. Gene–Diet Interactions Affecting Insulin Resistance and Metabolic Traits

The largest number of reported gene–diet interactions in this review involved insulin resistance and related metabolic traits, particularly in dietary intervention studies comparing weight-loss diets that differed in macronutrient composition. Several trials reported possible genotype-dependent responses to dietary fat or carbohydrate content. For example, *PPM1K* rs1440581 was associated with differential changes in insulin-related markers in response to low-fat versus high-fat diets [52,60], while *TCF7L2* rs7903146 and *IRS1* rs2943641 were linked to differential responses to diets varying in carbohydrate or fat composition [62,66]. Additional interaction signals were reported for loci such as *PCSK7*, *GIPR*, *FTO*, *HNF1A* and selected GRS, suggesting that insulin-related traits may be particularly sensitive to genotype-dependent variation in response to controlled dietary interventions.

A second group of studies suggested that genetic variation may also influence metabolic responses to dietary fat quality. Small crossover feeding trials reported differential insulin sensitivity responses to saturated, monounsaturated, or carbohydrate-rich diets according to variants in *APOE*, *FABP2*, *PPARγ*, *SCARB1*, *LIPC*, and other loci [68,73,76,78]. However, these studies were generally short in duration and small in sample size, and many findings have not been independently replicated.

Overall, this area provides some of the more promising signals in the review, particularly for short-term responses to weight-loss diets differing in macronutrient composition. Nevertheless, findings were not uniform across studies, and several reported interactions attenuated over longer follow-up [53]. This pattern suggests that any true genotype-dependent dietary effects may be modest, context-dependent, and sensitive to behavioral factors such as adherence and weight regain.

An additional consideration when interpreting findings from weight-loss interventions is that improvements in insulin sensitivity, glycemic control, and other metabolic outcomes are strongly influenced by weight reduction itself. Consequently, it may be difficult to disentangle the independent effects of dietary macronutrient composition or gene–diet interactions from the metabolic benefits associated with weight loss. This distinction is particularly important given evidence suggesting that weight loss is among the strongest determinants of improvements in T2D-related outcomes, often overshadowing differences attributable to dietary macronutrient composition alone [99,100]. Future nutrigenetic intervention studies should therefore carefully account for weight change, both as a potential confounder and as a mediator of genotype-dependent dietary responses.

### 4.3. Gene–Diet Interactions and Glycemic Traits

Evidence for gene–diet interactions affecting glycemic traits and β-cell-related outcomes was more limited than for incident T2D or insulin-related traits. Among the loci examined, *TCF7L2* emerged as the most repeatedly implicated, particularly in relation to macronutrient intake and selected food-based exposures. For example, Bauer et al. (2021) [91] reported that higher protein intake was associated with less favorable glycemic profiles among individuals carrying the *TCF7L2* rs7901695 TT genotype, including higher HbA1c and insulin responses during oral glucose tolerance testing. Similarly, Ouhaibi-Djellouli et al. (2014) [93] found that dessert consumption modified the association between *TCF7L2* rs7903146 and fasting glucose, with the T allele associated with higher fasting glucose only among frequent dessert consumers.

Beyond *TCF7L2*, the evidence was more fragmented. Ortega-Azorín et al. (2012) [95] reported an interaction between *FTO* rs9939609 and folate intake in relation to fasting glucose, while Tsuzaki et al. (2009) [97] and Enhörning et al. (2009) [96] described interactions involving *ADIPOQ* and *AVPR1A*, respectively. However, these findings were based on relatively few studies, involved different dietary exposures and outcomes, and have not been extensively replicated.

Taken together, the available evidence is compatible with the possibility that genetic variation influences glycemic responses to dietary exposures, although this area remains underdeveloped. In particular, β-cell-related outcomes and glycemic traits were examined in comparatively few studies, making it difficult to determine which reported interactions are robust.

### 4.4. Quality of Evidence

The overall quality of the evidence was limited by important methodological concerns across both randomized and non-randomized studies. Among the 30 randomized trials assessed with RoB 2, 19 (63.33%) were judged to be at high overall risk of bias and 11 (36.67%) were judged to raise some concerns. The domains contributing most frequently to these judgments were the randomization process, deviations from intended interventions, missing outcome data, and selection of the reported result, whereas measurement of outcomes was generally judged to be at low risk of bias.

Among the 31 non-randomized studies assessed with ROBINS-I, 28 (90.32%) were judged to have serious risk of bias, 2 (6.45%) critical risk, and 1 (3.23%) moderate risk. The most frequent concerns related to confounding, deviations from intended interventions or exposures, missing data, and selection of the reported result. In contrast, bias in measurement of outcomes was most often rated as low risk. Although ROBINS-I was originally developed for non-randomized studies of interventions, it provided a consistent framework for evaluating risk of bias across the heterogeneous non-randomized study designs included in this review. Given that several observational studies assessed habitual dietary exposures rather than assigned interventions, the resulting risk-of-bias assessments were interpreted cautiously and in the context of the methodological characteristics of each study design, consistent with current methodological guidance [34,35]. These findings indicate that, although several studies reported statistically significant gene–diet interactions, the overall body of evidence should be interpreted cautiously, particularly when findings were based on single studies or lacked independent replication.

Substantial heterogeneity in study design, dietary assessment methods, genetic modeling strategies, and outcome definitions further limits confidence in the consistency and interpretability of the findings. A formal GRADE assessment was not undertaken because the marked heterogeneity across study designs, exposures, outcomes, and analytical approaches also precluded meta-analysis.

Overall, the body of evidence differed across outcome domains. Evidence relating to incident T2D was largely derived from larger prospective studies, whereas evidence for insulin-related and glycaemic traits originated from a more heterogeneous body of observational and intervention studies that differed substantially in study design, dietary exposures, genetic variants, outcome assessment, and methodological quality. Consequently, confidence in individual gene–diet interactions remains limited, particularly where findings were based on single studies without independent replication and where substantial risk of bias was identified.

### 4.5. Methodological Heterogeneity Across Studies

A major characteristic of the included literature was the substantial methodological heterogeneity among studies investigating gene–diet interactions. This variability likely contributed to the inconsistency of reported findings and limited their comparability. First, study designs ranged from cross-sectional analyses and prospective cohort studies to controlled dietary intervention trials. Observational studies often relied on self-reported dietary intake, frequently assessed using food frequency questionnaires, which are susceptible to recall bias and measurement error. Such measurement error may attenuate gene–diet interactions, particularly when dietary exposures are broadly categorized [37,39]. In contrast, controlled feeding and dietary intervention trials generally provide more precise assessment of dietary exposures but are often limited by shorter intervention periods and smaller sample sizes. The inclusion of both observational and intervention studies enabled a more comprehensive evaluation of the available evidence on gene–diet interactions across clinically relevant metabolic outcomes. However, these study designs differ substantially in their methodological strengths and capacity to support causal inference. Accordingly, the findings were interpreted in the context of study design, methodological quality and risk of bias rather than statistical significance alone.

Second, statistical power varied substantially across studies. Detecting gene–diet interactions generally requires larger sample sizes than detecting main genetic or dietary effects. Although several cohort studies included large populations, many intervention studies enrolled fewer than 500 participants, which may have limited their ability to detect modest interaction effects.

Third, the genetic approaches used were not uniform. Some studies assessed single candidate SNPs, whereas others used genetic risk scores incorporating multiple loci. Although polygenic approaches may capture a greater proportion of genetic susceptibility, the composition and weighting of genetic risk scores varied considerably across studies, thereby limiting direct comparison across studies [40,65]. In addition, the genetic models used to evaluate interactions (e.g., additive, dominant, recessive, or allele-based models) differed considerably between studies and were often selected post hoc or inconsistently reported. This methodological variability further complicated comparison across studies and may have contributed to inconsistent findings.

Fourth, dietary exposure definitions also varied substantially. Macronutrient intake was expressed as a percentage of total energy, absolute intake, or broader dietary pattern scores, while dietary fat quality was represented by indices such as the polyunsaturated-to-saturated fat ratio, Mediterranean diet adherence, or controlled manipulation of fatty acid composition. These differences further complicate cross-study comparison.

Fifth, several studies evaluated total carbohydrate or total fat intake without adequately accounting for dietary quality. However, growing evidence suggests that carbohydrate source (e.g., whole grains and dietary fiber versus refined carbohydrates) and fat subtype (e.g., saturated, monounsaturated, and polyunsaturated fatty acids) may exert distinct effects on insulin sensitivity, glucose homeostasis, and T2D risk [101,102]. Consequently, dietary quality may be more biologically informative than macronutrient quantity alone when evaluating gene–diet interactions. Future Nutrigenetics studies should therefore incorporate more refined measures of dietary quality to improve comparability and enhance the biological interpretation of gene–diet interactions.

Moreover, study populations differed in ethnicity, age, metabolic status, and prevalence of obesity. Because allele frequencies, linkage disequilibrium patterns, and gene–environment interactions may vary substantially across populations, some reported interactions may be population-specific rather than broadly generalizable [80,81]. These differences also complicate replication across independent cohorts and may partly explain inconsistencies between studies.

Finally, multiple testing and limited independent replication remain important concerns. Many studies evaluated numerous dietary exposures and genetic variants without adequate correction for multiple comparisons or validation in independent cohorts, increasing the likelihood of false-positive findings. Collectively, these methodological limitations likely explain part of the inconsistency observed across studies and reinforce the need for larger, standardized, and independently replicated investigations.

Finally, an additional methodological limitation was the incomplete reporting of Hardy–Weinberg equilibrium (HWE). Thirteen studies did not report HWE, limiting the assessment of genetic data quality and contributing to uncertainty in the evaluation of study quality. In contrast, one study deviated from HWE, a finding that may reflect genotyping error, population stratification, or selection bias, and was therefore interpreted with greater caution.

### 4.6. Strengths and Limitations

A key strength of this review is the inclusion of both observational studies and dietary intervention trials, which broadens the scope of the evidence captured across diverse dietary exposures and metabolic outcomes. This comprehensive approach enhances the generalizability of findings and reflects the complexity of Nutrigenetics research. Reviews limited solely to RCTs may fail to capture long-term nutrient exposure effects or may overlook relevant findings due to the scarcity of trials available on certain gene–nutrient interactions. Including observational studies helps fill these evidence gaps and broadens the scope of analysis [32].

The inclusion of both randomized and observational studies was grounded in methodological considerations, acknowledging that randomization remains the most effective method to minimize confounding, whereas confounding can persist even in well-conducted observational research [103]. To ensure a robust evaluation of study quality, design-specific risk-of-bias assessment tools were employed. Specifically, the Cochrane Risk of Bias 2 (RoB 2) tool was used for randomized controlled trials, applying the versions developed for parallel-group and crossover designs as appropriate, whereas ROBINS-I was used for non-randomized intervention and observational studies [35]. This methodological framework enhances the transparency and consistency of quality assessment and facilitates a more balanced interpretation of the available evidence.

Several limitations should also be acknowledged. First, substantial heterogeneity in study design, dietary assessment methods, genetic modeling, outcome definitions, and statistical approaches precluded meta-analysis and limits comparability across studies. Second, many reported gene–diet interactions were based on single studies and lacked independent replication. Third, dietary assessment in observational studies was often based on self-reported intake, which is vulnerable to measurement error and may attenuate interaction effects. Fourth, the overall quality of the evidence was constrained by the high proportion of randomized trials at high risk of bias and non-randomized studies at serious risk of bias. Fifth, only full-text, peer-reviewed articles published in English were included, while gray literature (e.g., conference abstracts, dissertations, trial registries, and preprints) was not searched. This may have introduced language and publication bias by underrepresenting unpublished, non-significant, or less prominent findings [32]. Consequently, this review may have disproportionately captured studies with more prominent or statistically significant findings, potentially underrepresenting smaller or non-significant effects [104]. Finally, a formal assessment of reporting bias and a structured certainty-of-evidence assessment (e.g., GRADE) were not undertaken because of the substantial methodological and clinical heterogeneity of the included studies. Although these approaches were not considered feasible in the context of the available evidence, their absence represents an additional limitation and should be considered when interpreting the overall body of evidence.

### 4.7. Mechanistic Considerations and Future Directions in PN Research

PN offers a biologically plausible framework for improving the prevention and management of T2D because metabolic responses to diet are shaped by substantial interindividual variability, including variability related to genetic background. More broadly, PN aims to tailor dietary advice using combinations of genetic, metabolic, behavioral, and environmental information, and has been proposed as a promising strategy for chronic disease prevention and management, including T2D [105,106].

In the context of T2D, there is a reasonable premise for the use of PN because many loci implicated in gene–diet research map to pathways involved in insulin secretion, insulin signaling, adiposity, lipid metabolism, and postprandial glycemic regulation. At the same time, recent reviews emphasize that nutrigenetic and genotype-based PN strategies remain at an early translational stage: biological plausibility is strong, but the evidence base is still limited by heterogeneous study designs, small or selected samples, insufficient ancestral diversity, and a lack of robust independent replication.

Beyond genetic influences, evidence from major lifestyle intervention trials has consistently demonstrated that dietary modification, weight loss, and increased physical activity can substantially reduce T2D risk, irrespective of genetic predisposition [100]. Although the present review focused on gene–diet interactions, physical activity represents another key determinant that may modify metabolic responses to dietary interventions [99]. Several studies included in this review adjusted their analyses for physical activity; however, assessment methods varied considerably, and structured exercise interventions were not the primary focus of the included studies. Future Precision Nutrition studies should therefore consider integrated gene–diet–physical activity interactions using standardized measures of physical activity and cardiorespiratory fitness. More recent evidence further suggests that individuals at elevated genetic risk may derive comparable or even greater benefit from lifestyle interventions [99]. Therefore, genetic susceptibility should not be viewed as a determinant of disease inevitability, but rather as one factor that may contribute to interindividual variability in metabolic responses to dietary exposures [107]. Within this framework, PN approaches aim to refine and optimize established lifestyle recommendations rather than replace them.

These considerations also highlight several priorities for future PN research. Rather than simply identifying additional candidate interactions, future studies should focus on generating replicable and clinically interpretable evidence. Future studies should be adequately powered, prospectively designed, and include prespecified interaction models, harmonized dietary exposures and metabolic outcomes, and replication across independent and ethnically diverse populations. Greater integration of genomics with other layers of information—such as metabolomics, microbiome data, continuous glucose monitoring, and behavioral phenotyping—may also improve the prediction of individual dietary responses [25,105,108].

At the same time, future PN strategies should not focus exclusively on individual gene–nutrient interactions. Increasing evidence supports the importance of whole dietary patterns, particularly plant-based diets, as effective non-pharmacological strategies for improving insulin sensitivity and reducing the risk of T2D. Their beneficial effects are thought to arise from the combined action of dietary fiber, unsaturated fatty acids, antioxidants, and phytochemicals, which collectively influence multiple pathways involved in glucose homeostasis and insulin sensitivity [109,110]. As PN continues to evolve, future approaches should move beyond individual gene–nutrient interactions and integrate evidence-based dietary patterns with genetic, metabolic, behavioral, and other biological information to provide more comprehensive and individualized prevention strategies. Complementary interventions—including functional foods, nutraceuticals, and, where clinically appropriate, pharmacological therapies—may further enhance the implementation of PN; however, robust evidence supporting their interaction with genetic susceptibility remains limited and warrants further investigation.

Another promising avenue for future PN research involves the gut microbiota. Interactions between host genetics suggest that the gut microbiota represents an important mediator of individual metabolic responses to dietary interventions. Interactions between host genetics, dietary exposures, and gut microbial composition may influence glucose homeostasis, insulin sensitivity, inflammatory pathways, and the production of bioactive metabolites, thereby contributing to interindividual variability in metabolic outcomes [111,112]. Consequently, integrating gut microbiome profiling with genomic and metabolic data may further enhance the accuracy and clinical applicability of PN. However, none of the studies included in the present review incorporated microbiome assessments, highlighting an important gap that should be addressed in future Nutrigenetics research.

Overall, the findings of this review indicate that PN and Nutrigenetics hold considerable promise for T2D prevention and management, but the evidence remains insufficient to support routine clinical implementation. At present, the field is supported more strongly by biological rationale and early proof-of-concept studies than by consistently replicated intervention evidence [21]. Translation into routine clinical practice will ultimately depend on the generation of robust, reproducible, and clinically meaningful evidence demonstrating that genotype-informed or multi-omic dietary strategies provide benefits beyond current evidence-based dietary recommendations. Particular emphasis should be placed on the independent replication of previously reported gene–diet interactions, especially those involving frequently studied loci such as *TCF7L2*, *IRS1*, *GIPR*, *FTO*, and polygenic risk scores. Establishing the reproducibility and clinical relevance of these interactions across diverse populations will be essential before their incorporation into routine Precision Nutrition strategies can be justified.

## 5. Conclusions

This systematic review identified 61 reports examining gene–diet interactions in relation to incident T2D, insulin-related traits and glycemic outcomes among adults without diagnosed T2D at baseline. Reported interactions involved a range of dietary exposures and loci, most commonly *TCF7L2*, *IRS1*, *GIPR*, *ADIPOQ* and *FTO*.

However, the evidence base was heterogeneous in design, exposure assessment, outcome definition and analytical approach, and many reported interactions were based on single studies without independent replication. In addition, the overall quality of the evidence was limited by substantial risk of bias in many included studies.

Although numerous statistically significant gene–diet interactions were reported, confidence in individual findings should be interpreted in light of the corresponding risk-of-bias assessments, with many studies judged to be at high or serious risk of bias. Taken together, the literature supports the biological plausibility of gene–diet interactions in T2D-related phenotypes, but robust, reproducible, and clinically actionable findings remain limited. Future research should prioritize larger and more diverse populations, standardized dietary assessment, prespecified analytical strategies, and independent replication. At present, the available evidence does not support the routine use of genotype-based dietary recommendations for T2D prevention outside research settings or carefully validated clinical contexts.

## Figures and Tables

**Table 1:** Eligibility criteria based on the PICOS framework.

Component	Criterion
Population (P)	Adults without diagnosed T2D at baseline, with available genetic and dietary exposure data.
Intervention/Exposure (I)	Dietary exposures including (a) habitual nutrient or food intake (e.g., macronutrients, dietary patterns, specific foods), and/or (b) nutrient supplementation. All studies included genotyping for polymorphisms in genes related to glucose homeostasis, insulin resistance, or T2D risk, reported as individual SNPs, haplotypes, or genetic risk scores.
Comparator (C)	(a) Different levels or categories of dietary intake (e.g., high vs. low intake), different doses of nutrient supplementation, or placebo; and (b) genetic comparisons, including variant vs. ancestral alleles, different genotype groups, haplotypes, or categories of genetic risk scores.
Outcomes (O)	Outcomes related to glucose metabolism, including incident T2D, fasting glucose, HbA1c, insulin, HOMA-IR, HOMA-B, OGTT-derived measures, and other markers of insulin resistance or β-cell function.
Study design (S)	Observational studies (cohort, case–control, cross-sectional) and intervention studies (randomized controlled trials, crossover trials, non-randomized interventions) examining gene–diet interactions.

HbA1c, Glycated hemoglobin; HOMA-B, Homeostatic Model Assessment of β-cell function; HOMA-IR, Homeostatic Model Assessment for Insulin Resistance; OGTT, Oral glucose tolerance test.

## Data Availability

The database search strategies are provided in the Supplementary Materials. The datasets generated and analyzed during the current study are available from the corresponding author upon request.

## Supplementary Materials

The following supporting information can be downloaded at: https://www.mdpi.com/article/10.3390/nu18152439/s1, Table S1: Search Strategies; Table S2: PRISMA 2020 checklist.

## Abbreviations

The following abbreviations are used in this manuscript:

DASH	Dietary Approaches to Stop Hypertension
EPIC	European Prospective Investigation into Cancer and Nutrition
GRS	Genetic Risk Score
GWAS	Genome-Wide Association Studies
HWE	Hardy–Weinberg equilibrium
IGT	Impaired Glucose Tolerance
PICOS	Population, Intervention/Exposure, Comparator, Outcome, Study design
PN	Precision Nutrition
PREDIMED	Prevención con Dieta Mediterránea
PRISMA	Preferred Reporting Items for Systematic Reviews and Meta-Analyses
PROSPERO	International Prospective Register of Systematic Reviews
RCT	Randomized Controlled Trial
RoB 2	Revised Cochrane risk-of-bias tool for randomized trials
ROBINS-I	Risk Of Bias In Non-randomized Studies of Interventions
ROBVIS	Risk of Bias Visualization
SNP	Single Nucleotide Polymorphism
T2D	Type 2 diabetes
