# Supplementary material for: Gene–Diet Interactions in Type 2 Diabetes: A Systematic Review of Observational Studies and Clinical Trials
Source: Nutrients. 2026 Jul 26;18(15):2439. doi: 10.3390/nu18152439 (PMC13467785; doi:10.3390/nu18152439)
Supplement: Supplementary file 1 [file nutrients-18-02439-s001.zip › nutrients-4420936-supplementary Table S1.pdf]

Table S1: Search Strategies

Search	Search Terms
1	"Diet"[MeSH Terms]
2	"Diet Therapy"[MeSH Terms]
3	"Dietary Supplements"[MeSH Terms]
4	"Dietary Patterns"[MeSH Terms]
5	"Nutrients"[MeSH Terms]
6	diet*[Title/Abstract]
7	nutrient*[Title/Abstract]
8	nutriti*[Title/Abstract]
9	supplement*[Title/Abstract]
10	macronutrient*[Title/Abstract]
11	micronutrient*[Title/Abstract]
12	"eating pattern*"[Title/Abstract]
13	"fat intake"[Title/Abstract] OR "dietary fat*"[Title/Abstract] OR carbohydrate*[Title/Abstract] OR protein*[Title/Abstract]
14	fiber[Title/Abstract] OR fibre[Title/Abstract] OR wholegrain*[Title/Abstract] OR "whole grain*"[Title/Abstract]
15	#1 OR #2 OR #3 OR #4 OR #5 OR #6 OR #7 OR #8 OR #9 OR #10 OR #11 OR #12 OR #13 OR #14
16	"Polymorphism, Genetic"[MeSH Terms]
17	"Polymorphism, Single Nucleotide"[MeSH Terms]
18	"Genotype"[MeSH Terms]
19	"Haplotypes"[MeSH Terms]
20	"Genetic Predisposition to Disease"[MeSH Terms]
21	gene*[Title/Abstract]
22	geno*[Title/Abstract]
23	polymorphism*[Title/Abstract]
24	varia*[Title/Abstract]
25	haplotype*[Title/Abstract]
26	#16 OR #17 OR #18 OR #19 OR #20 OR #21 OR #22 OR #23 OR #24 OR #25
27	"Diabetes Mellitus, Type 2"[MeSH Terms]

28	"Prediabetic State"[MeSH Terms]
29	"Insulin Resistance"[MeSH Terms]
30	"Hyperglycemia"[MeSH Terms]
31	"Blood Glucose"[MeSH Terms]
32	"Glucose Intolerance"[MeSH Terms]
33	"Hemoglobin A, Glycosylated"[MeSH Terms]
34	"Insulin Secretion"[MeSH Terms]
35	"diabet*"[Title/Abstract] OR T2D[Title/Abstract]
36	"insulin"[Title/Abstract]
37	"glucose"[Title/Abstract]
38	glyc*[Title/Abstract]
39	"HbA1c" [Title/Abstract]
40	"beta-cell"[Title/Abstract]
41	"β-cell"[Title/Abstract]
42	HOMA*[Title/Abstract]
43	#27 OR #28 OR #29 OR #30 OR #31 OR #32 OR #33 OR #34 OR #35 OR #36 OR #37 OR #38 OR #39 OR #40 OR #41 OR #42
44	#15 AND #26 AND #43
45	"animals"[MeSH Terms]
46	"humans"[MeSH Terms]
47	#45 NOT #46
48	#44 NOT #47
